# Neural Connectivity Changes Facilitated by Familiar Auditory Sensory Training in Disordered Consciousness: A TBI Pilot Study

**DOI:** 10.3389/fneur.2020.01027

**Published:** 2020-10-08

**Authors:** Theresa L. Bender Pape, Sherri L. Livengood, Sandra L. Kletzel, Brett Blabas, Ann Guernon, Dulal K. Bhaumik, Runa Bhaumik, Trudy Mallinson, Jennifer A. Weaver, James P. Higgins, Xue Wang, Amy A. Herrold, Joshua M. Rosenow, Todd Parrish

**Affiliations:** ^1^The Department of Veterans Affairs (VA), Center for Innovation in Complex Chronic Healthcare & Research Service, Edward Hines Jr. VA Hospital, Hines, IL, United States; ^2^Department of Physical Medicine and Rehabilitation, Northwestern University Feinberg School of Medicine, Chicago, IL, United States; ^3^Marianjoy Rehabilitation Hospital Part of Northwestern Medicine, Wheaton, IL, United States; ^4^Division of Epidemiology and Biostatistics, Department of Psychiatry, Biostatistical Research Center, University of Illinois at Chicago, Chicago, IL, United States; ^5^Research Service, Cooperative Studies Program Coordinating Center, Edward Hines Jr. VA Hospital, Hines, IL, United States; ^6^Department of Clinical Research and Leadership, School of Medicine and Health Sciences, The George Washington University, Washington, DC, United States; ^7^Department of Radiology, Northwestern University Feinberg School of Medicine, Chicago, IL, United States; ^8^Department of Psychiatry and Behavioral Sciences, Feinberg School of Medicine, Northwestern University, Chicago, IL, United States; ^9^Department of Neurological Surgery, Northwestern University Feinberg School of Medicine, Chicago, IL, United States; ^10^Northwestern Memorial Hospital, Chicago, IL, United States

**Keywords:** attention, consciousness disorders, language, traumatic brain injury, white matter

## Abstract

For people with disordered consciousness (DoC) after traumatic brain injury (TBI), relationships between treatment-induced changes in neural connectivity and neurobehavioral recovery have not been explored. To begin building a body of evidence regarding the unique contributions of treatments to changes in neural network connectivity relative to neurobehavioral recovery, we conducted a pilot study to identify relationships meriting additional examination in future research. To address this objective, we examined previously unpublished neural connectivity data derived from a randomized clinical trial (RCT). We leveraged these data because treatment efficacy, in the RCT, was based on a comparison of a placebo control with a specific intervention, the familiar auditory sensory training (FAST) intervention, consisting of autobiographical auditory-linguistic stimuli. We selected a subgroup of RCT participants with high-quality imaging data (FAST *n* = 4 and placebo *n* = 4) to examine treatment-related changes in brain network connectivity and how and if these changes relate to neurobehavioral recovery. To discover promising relationships among the FAST intervention, changes in neural connectivity, and neurobehavioral recovery, we examined 26 brain regions and 19 white matter tracts associated with default mode, salience, attention, and language networks, as well as three neurobehavioral measures. Of the relationships discovered, the systematic filtering process yielded evidence supporting further investigation of the relationship among the FAST intervention, connectivity of the left inferior longitudinal fasciculus, and auditory-language skills. Evidence also suggests that future mechanistic research should focus on examining the possibility that the FAST supports connectivity changes by facilitating redistribution of brain resources. For a patient population with limited treatment options, the reported findings suggest that a simple, yet targeted, passive sensory stimulation treatment may have altered functional and structural connectivity. If replicated in future research, then these findings provide the foundation for characterizing the unique contributions of the FAST intervention and could inform development of new treatment strategies. For persons with severely damaged brain networks, this report represents a first step toward advancing understanding of the unique contributions of treatments to changing brain network connectivity and how these changes relate to neurobehavioral recovery for persons with DoC after TBI.

**Clinical Trial Registry:** NCT00557076, The Efficacy of Familiar Voice Stimulation During Coma Recovery (http://www.clinicaltrials.gov).

## Introduction

Coma recovery after traumatic brain injury (TBI) is described by degrees of consciousness delineated clinically as the vegetative state (VS), minimally conscious state (MCS), and emergence from MCS (1–4). These classifications represent a gradient of clinical consciousness where less consciousness is associated with more disruption of functional and structural neural connectivity (5–18). Recovery, however, is not necessarily a linear progression along this gradient (10, 19–21). Cross-sectional evidence suggests that behavioral recovery is supported by dynamic changes in hypoconnectivity and hyperconnectivity of neural networks local to and remote from lesion topography (8, 22–28). Emerging longitudinal evidence (29) also suggests that behavioral recovery (30) is supported by non-linear changes in hyporesting and hyperresting state functional neural connectivity (10, 19, 20, 31, 32). Collectively, the evidence suggests that changes in neural connectivity could precede or occur in parallel with neurobehavioral recovery.

Advancing knowledge of how changes in neural connectivity relate to neurobehavioral recovery is important, in part, because it will allow for identification of the unique contributions of specific interventions to brain and behavior relationships. To start building a body of evidence regarding the contributions of specific interventions to changes in neural network connectivity and the relationship of these changes to neurobehavioral recovery, we conducted a *post hoc* pilot study using a systematic approach to examine previously unpublished neural connectivity data from a double-blind randomized clinical trial (RCT) (33). As this RCT demonstrated the therapeutic efficacy of the familiar auditory sensory training (FAST) intervention, this longitudinal dataset enabled an examination of the unique contributions of a specific intervention, the FAST. Considering that usual care was paired, by random assignment, with either the FAST intervention or the placebo intervention, the RCT dataset also provides the basis to identify changes in neural connectivity specific to the FAST vs. changes related to differences in usual care practices (34, 35) and/or placebo effects (36). The RCT design also allows for the accounting of injury heterogeneity (etiologies, neuropathology, and secondary brain damage) in measures of change in neural connectivity.

**Figure 1:**
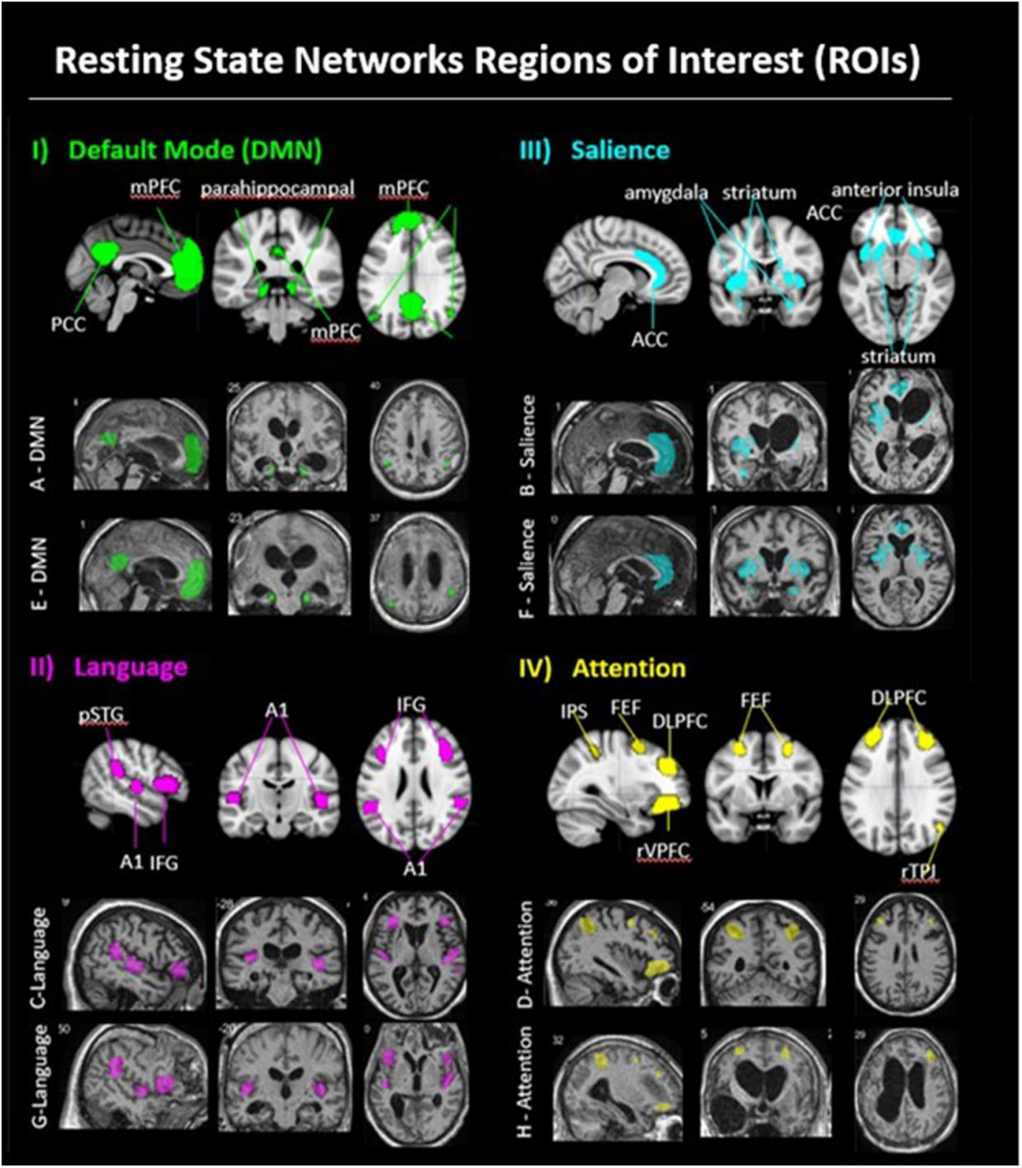
Resting state networks regions of interest (ROI). (i) DMN: Panel I, row 1 shows a DMN exemplar on the MNI 152 standard brain, with medial prefrontal cortex (mPFC), posterior cingulate cortex (PCC), bilateral parahippocampal area, bilateral temporal parietal junction (TPJ). Panel I, row two shows example of a FAST subject's DMN ROIs (patient A); panel I. row 3 shows an example of a placebo subject's DMN ROIs (patient E). (ii) Language: Panel II, row 1 shows the language network ROIs on the MNI 152 standard brain, with bilateral posterior superior temporal gyrus (pSTG; Wernicke area), bilateral primary auditory cortex (Heschl gyrus), and bilateral inferior frontal gyrus (IFG; Broca area). Panel II, row 2 shows an example of a FAST subject's language ROIs (patient C); panel II, row 3 shows an example of a placebo subject's language ROIs (patient G). (iii) Salience: Panel III, row 1 shows the salience network ROIs on the MNI 152 standard brain, with anterior cingulate cortex (ACC), bilateral amygdala, bilateral anterior insula, and bilateral striatum. Panel III, row 2 shows an example of a FAST subject's salience ROIs (patient B); panel III, row 3 shows an example of a placebo subject's salience ROIs (patient F). (iv) Attention: Panel IV, row 1 shows the attention network ROIs on the MNI 152 standard brain, including bilateral dorsal lateral prefrontal cortex (DLPFC), bilateral frontal eye fields (FEF), bilateral intraparietal sulcus (IPS), right temporal parietal junction (rTPJ), and right ventral lateral prefrontal cortex (rVLPFC). Panel IV, row 2 shows an example of a FAST subject's attention ROIs (patient D); panel IV, row 2, shows an example of a Placebo subject's attention ROIs (patient H).

The purpose of this article is to report pilot study findings of changes in neural connectivity related to the FAST intervention for persons remaining in states of disordered consciousness (DoC) after TBI. To the best of our knowledge, for this population, this article represents the first report of longitudinally based profiles of neural network connectivity changes relative to neurobehavioral recovery in response to a specific therapeutic intervention. For this scientifically challenging patient population, the reported study also demonstrates a systematic approach to explicating the relationships among neurobehavioral recovery and changes in neural connectivity of the broad neural networks thought to be targeted by the autobiographical auditory-linguistic stimuli used in the FAST intervention (37) (also see Supplement A): the language network (LN), salience network (SN), attention network (AN), and default mode network (DMN) (Figure 1). The reported findings and scientific approach used to discover and identify relationships warranting further study, together, provide guidance for future research examining the unique contributions of the FAST intervention, as well as other interventions to neurobehavioral recovery from DoC after TBI.

## Materials and Methods

The present study is based on data for a subgroup of FAST RCT participants. The previously published RCT methods (33) relevant to the present study as well as procedures unique to the present study are provided here. The RCT was approved by the human subjects' institutional review boards at each study site. Informed consent was obtained from each participant's legally authorized representative.

The imaging subgroup was selected from the total FAST RCT sample. RCT participants were recruited from a Veterans Administration Inpatient Polytrauma Rehabilitation program, the Shirley Ryan Ability Lab inpatient rehabilitation program, and from community residences in a large urban area [see Pape et al. (33) for additional details]. To be eligible for the RCT, participants were required to (a) be 18 years or older, (b) have incurred a severe TBI within the previous year, and (c) be in a state of DoC for at least 28 days as a result of the TBI. Persons dependent on a ventilator and remaining in states of DoC due to non-traumatic or penetration injuries were not eligible. Randomization was stratified by each of the three study settings. Sixteen participants were randomized to the FAST or placebo groups, 15 of whom completed the RCT [mean age at injury = 35.1, standard deviation (SD) = 11.0; mean days after TBI = 69.8, SD = 42.8; male = 80%; MCS = 67%].

### Neurobehavioral Outcomes

The primary and secondary neurobehavioral outcomes for the FAST RCT were the Disorders of Consciousness Scale-25 (DOCS-25) (38) and the Coma–Near-Coma (CNC) scale (39), respectively. As summarized in Supplement C, the DOCS-25 yields a reliable and valid measure (40) of best global overall multimodal neurobehavioral functioning, and it includes an Auditory-Language subscale. As this subscale measure was not the primary or secondary RCT outcome, the DOCS-25 Auditory-Language subscale measures were not previously reported (33). These subscale measures were included in the present study because they are specific and relevant to neural networks involved in language function and processing thought to be targeted with the FAST stimuli (37) (also see Supplement A). The CNC total score is a measure of arousal, attention, and awareness (41–43); lower scores reflect more consistent behavioral responses (39, 44).

Baseline (BL) and endpoint (EP) neurobehavioral tests were obtained 24 h after complete cessation of pharmaceutical CNS stimulants for all participants. The DOCS-25 was repeated weekly, and the CNC was repeated twice weekly (also see Figure SC1 in Supplement C).

### Image Acquisition

Anatomical, resting state, and diffusion tensor imaging (DTI) data were acquired at BL (prior to starting assigned intervention) and again at treatment EP. For this multisite RCT, all scans were acquired on two different 1.5-T scanners with a standard 12-channel head coil. Foam cushions and earplugs were used to reduce motion and scanner noise. Scanners were cocalibrated using previously described procedures [see Supplementary Material in Pape et al. (33)]. For the present study, however, all participants were scanned on the same magnet (i.e., Siemens Avanto).

For functional magnetic resonance imaging (MRI) resting state acquisition, 205 whole-brain T2^*^-weighted echo planar images (EPIs) were acquired in a 10-min, 15-s scan, with a repetition time (TR) of 3 s; echo time (TE) of 40 ms; and a flip angle of 90°, with a 64 × 64 matrix, a field of view (FOV) = 220 × 220 mm, and a voxel size of 3.4 × 3.4 × 3.0 mm^3^. For registration purposes, a high-resolution anatomical T1-weighted sequence was acquired at BL and, considering potential morphological changes following treatment, was also collected at EP. Both were collected using three-dimensional (3D) magnetization-prepared rapid gradient-echo sequence with the following parameters: 176 slices, TR/TE/flip angle = 2,400 ms/3.72 ms/8°, inversion time (TI) = 1,000 ms, voxel size = 1 × 1 × 1 mm^3^, FOV = 256 mm.

DTI data were acquired using a spin-echo EPI sequence with the following parameters: TR/TE/flip angle = 6,000 ms/89 ms/90°; 30 gradient orientations with *b* = 1,000 s/mm^2^, three images with *b* = 0, FOV = 220 × 220 mm with a matrix size of 144 × 120 leading to an in-plane resolution of 1.77 × 1.77 × 3 mm^3^.

### Functional Imaging Preprocessing

Resting state EPI data were preprocessed using SPM8 in MATLAB R2012b. The first three volumes of the 205 acquired were discarded for MRI signal stabilization. The remaining 202 volumes were realigned to the first EPI volume. To preserve anatomical injuries and reduce spatial variability between subjects to enable group comparisons, the voxel-based morphometry toolbox was used for the non-linear warping to normalize the best T1 image using DARTEL (Diffeomorphic Anatomical Registration Through Exponentiated Lie algebra; see Figure SC2 in Supplement C for non-linear warping explanation and illustration) from BL and EP to the Montreal Neurological Institute 152 template (MNI152). BL and EP resting state data were linearly warped to the native T1 using SPM. Because of lesion type and location variability between subjects, a region of interest (ROI)–based manual segmentation was performed for each participant. Blood oxygen level–dependent (BOLD) data were detrended and bandpass filtered (0.01–0.08 Hz). The white matter mask for resting state data covers normal-appearing white matter superior to the lateral ventricle. The cerebral spinal fluid mask for resting state data covers the middle section of the lateral ventricle farthest from the gray matter boundary. While global signal regression has been shown to reduce motion effects in resting state data, applying global signal regression is controversial as it has also been shown to remove some valuable neural signal and to alter the BL reference (45). Therefore, we ran a binary regression on each volume with motion regressor thresholds set to the temporal derivative of the time course (DVARS, where VARS = RMS of the variance over voxels), for DVARS > 50 (i.e., 5% of BOLD signal) and framewise displacement (FD < 0.5 mm) as thresholds (46). Following the binary regression, one participant had only 108 remaining resting state volumes. To enhance comparability between subjects, for each subject the first 108 volumes that survived the binary regression thresholds were used for analyses (≈5 min and 24 s of resting state data per subject).

### DTI Preprocessing

DTI data were processed using the FMRI Software Library (FSL) Diffusion Toolkit (FDT; http://www.fmrib.ox.ac.uk/fsl). The brain extraction tool was applied to the first non-diffusion-weighted B0 image for skull stripping, and a mask was created from the skull-stripped volume. FDT diffusion was used for eddy current and motion correction. DTIFIT was used to create voxelwise diffusion tensor models within the whole-brain mask to produce fractional anisotropy (FA) maps. The longitudinal data were not normalized to a standard template. FLIRT utilities were used to create halfway transforms to apply the same level of transformation (interpolation) to BL and EP data for each subject. The EP b0 image was registered to the BL b0 image using an Affine 12 parameter model. The command “avscale” was run on the EP-to-BL registration output matrix to generate a “halfway forward” and “halfway backward” transformation matrix. The halfway backward transform was then applied to EP FA maps, and the halfway forward transform was applied to the BL FA maps. FSLmaths was used to binarize and multiply the FA maps from both timepoints, to produce a mask of common voxels, with a threshold of 0.2 to include partial-volume edges.

### Imaging Subgroup Selection

The FAST RCT included a total of 15 participants, with 14 being scanned on the same magnet (i.e., Siemens Avanto). Of these 14 participants, six were excluded from the imaging subgroup. The primary reason for exclusion was motion that, after motion reduction procedures conducted during preprocessing, exceeded 3 mm (also see consort diagram, Supplement B). For the remaining eight participants, motion was also examined according to within-subject differences between BL and EP. To identify motion differences, we conducted a series of two-tailed non-parametric Wilcoxon signed rank tests for paired BL and EP measures of the FD and the temporal derivative of the time course (DVARS, where VARS = RMS of the variance over voxels) and found no significant differences [FAST FD BL (*n* = 4) mean = 0.42, SD = 0.36 vs. EP (*n* = 4) mean = 0.66, SD = 0.30, *p* = 0.10; FAST DVARS BL (*n* = 4) mean = 37.73, SD = 6.35 vs. EP (*n* = 4) mean = 36.27, SD = 6.41, *p* = 0.86; placebo FD BL (*n* = 4) mean = 0.66, SD = 0.40 vs. EP (*n* = 4) mean = 0.78, SD = 0.65, *p* = 0.86; placebo DVARS BL (*n* = 4) mean = 53.03, SD = 15.01 vs. EP (*n* = 4) mean = 41.93, SD = 10.23, *p* = 0.36]. Thus, the imaging subgroup for the present study includes eight RCT participants scanned on the same magnet with imaging data of sufficient quality (*n* = 4 from FAST group and *n* = 4 from the placebo group; Table 1).

**Table 1:** Pilot study imaging Subgroup demographics.

	**Total (*n =* 8)**	**FAST (*n =* 4)**	**Placebo (*n =* 4)**
Mean age at injury (years)	39.6, SD = 12.3	40.5, SD = 13.7	38.8, SD = 12.7
Mean days after TBI	77.9, SD = 37.6	77.8, SD = 38.2	78.0, SD = 42.9
Percent male	0.88	0.75	100
Percent VS baseline	0.38	0.50	0.25
Percent MCS baseline	0.62	0.50	0.75
Percent VS endpoint	0.25	0.25	0.25
Percent MCS endpoint	0.50	0.25	0.75
Percent eMCS endpoint	0.25	0.50	0.00

*MCS, minimally conscious state; eMCS, emerged from MCS to full consciousness; SD, standard deviation; VS, vegetative state*.

### Regions of Interest

For the eight participants in the present study, resting state functional connectivity (rsFC) analyses were conducted using a total of 26 ROIs, which were manually drawn by a single researcher on the normalized T1 (Figure 1) in MRICron (47). Regions were referenced against gray matter atlases (48, 49). For six of the eight participants, ROIs were drawn on the BL normalized T1 and applied to the coregistered data at EP. The two remaining participants, however, had substantial morphological changes at EP (i.e., reduced subdural hematoma; cranioplasty); thus, ROIs were separately hand-drawn for those two participants on the normalized EP T1 and BL normalized T1.

Given anatomical variability between subjects, an ROI-based manual segmentation method was also implemented for DTI analyses. All 19 white matter tracts were hand-drawn for each participant by a single researcher. To minimize error and to exploit fiber orientation, tracts were drawn on each coronal slice of the BL 2D FA maps using the b0 images as the primary anatomical reference. Each tract was drawn in its entirety, from the largest to the smallest diameter sections, while being referenced against the ICBM-DTI-81 white-matter labels atlas in FSL. ROI verification and refinements were made in the sagittal and axial planes (50, 51). The whole-brain mask of common voxels was then multiplied by each white matter ROI to isolate ROI specific voxels, present at both timepoints.

### Computation of Brain Network Connectivity Metrics

To compute rsFC metrics, a time series of the resting state BOLD signal was extracted for each of the 26 ROIs using the MATLAB REST toolbox. If an ROI was not discernible or not present, then the time series was not imputed and was classified as missing data. For rsFC network analyses, we then used two distinct approaches to compute brain connectivity metrics indicative of communication within-networks and between-networks. For within-network analyses, we computed rsFC metrics using an approach that preserves ROI–ROI correlation strengths. For between-network connectivity analyses, we computed rsFC metrics using a network masking approach that preserves signal strength.

To address the potential for broad dysregulation within an individual network, we used an ROI–ROI correlation approach to compute the rsFC metrics for use in within-network analyses. This approach preserves correlation strengths across each ROI including the anticorrelations and differences in ROI–ROI correlation strengths. Specifically, this approach uses ROI–ROI correlation strengths to characterize the synchrony within an individual network.

After generating a 26 × 26 pairwise ROI–ROI correlation matrix, (52) the Fisher *z* transformation was applied. We calculated rsFC of each individual network (DMN, LN, SN, AN) by averaging the ROI–ROI Pearson correlations *z* scores within a network and generated within-participant and group-level (FAST, placebo) means.

To assess between-network connectivity, we used an approach to computing rsFC metrics that preserves signal strength (rather than correlation strength) to weigh the contribution of each node. This analytic strategy yields a better reflection of between-network communication where any strong node can drive the network–network communication. For each network, a mask was created by averaging the time series for each ROI (i.e., rather than averaging individual ROI–ROI correlations). A distinct advantage of this approach is that it preserves signal strength (over correlation strength), thereby preserving the contribution of dominant nodes. Thus, the use of signal strength as an index of between-network communication allows for any strong node to drive the network–network communication. For example, a very strong signal may be anticorrelated with a very weak signal; the correlation is strong, but the contribution from each node is unequal. Conversely, two weak anticorrelated signals may produce similar correlation strength. Averaging the signals preserves the contribution of the dominant signal while averaging out the weak anticorrelations.

To calculate between-network rsFC, the time series for each ROI in a network was extracted and then averaged at the network level (e.g., for LN: L Broca + R Broca + L Heschl + R Heschl + L Wernicke + R Wernicke/6). The resulting network time series for the DMN, LN, SN, and AN were then used to generate a Fisher *z*-transformed 4 × 4 correlation matrix for each participant. Group-level (FAST, placebo) means were calculated.

For DTI metrics, we examined FA that is a reliable metric that self-normalizes between 0 and 1 (53). We used fslstats in the FSL toolkit to extract mean FA values and SDs for each ROI. Group-level (FAST, placebo) mean FA values were then calculated for each timepoint (54).

### Computing Neurobehavioral and Neural Connectivity Measures for Use in Data Analyses

The raw neurobehavioral scores and brain connectivity metrics were used to compute equal-interval measures according to a process of precision optimization. For neurobehavioral measures, this computational process started with transforming raw scores using Rasch measurement models because this enhances precision of each patient's estimated neurobehavioral function (55, 56). The DOCS-25 Total and DOCS-25 Auditory-Language subscale scores were transformed using multifaceted Rasch measurement (MFRM) (57–59). The MFRM approach enhances measurement precision by neutralizing between-rater differences and by attenuating within- and between-subject variability not related to neurobehavioral function (40, 57–59). Because CNC test items each have a unique rating scale, CNC raw scores were transformed using a partial-credit Rasch model (60) as it accounts for differing scales.

The Rasch transformed DOCS-25 Total, DOCS-25 Auditory-Language, and CNC measures and all brain network connectivity metrics were used in mixed-effects linear models (MLMs). The MLM approach was used because it produces robust parameter estimates by borrowing strength from each and every measure, accounting for interdependency of repeated observations and by accounting for within- and between-subject variability (61). Specifically, MLM was used to estimate model parameters, the intercept (β_1_), slope (β_2_), and both random effects (see Supplement C for additional details on MLM modeling procedures).

For each MLM of the DOCS-25 Total, DOCS-25 Auditory-Language, and CNC measures, the precision of MLM model parameter estimates was enhanced by including the Rasch transformed neurobehavioral measures for all 15 RCT participants (62). For the DOCS-25 Total and DOCS-25 Auditory-Language models, each MLM included six timepoints per participant. For the CNC model, 12 timepoints per participant were included. After MLM, the neurobehavioral measures for the eight participants in the imaging subgroup were extracted from MLM results and retained for data analyses.

For MLM of the brain network connectivity metrics, each of the eight subgroup participants was used in the models. Fisher *z*-transformed metrics for each gray matter ROI and BL mean FA metrics were used in MLM (62). Two timepoints (BL and EP) were included in each model.

All MLM-derived neurobehavioral and brain connectivity measures, prior to conducting analyses, were verified by computing mean absolute error (MAE) and mean absolute relative error (MARE). The MAE and MARE identify the average error of the MLM estimated measures by BL, EP, and change from BL to EP.

### Accounting for Missing Substrates in Brain Network Connectivity Metrics

Because of the uniqueness of the neuropathology among participants, each individual's own normalized image was used to represent their brain injury. Using this approach, the Fisher *z*-transformed values followed an approximately normal distribution. However, each participant had at least one gray matter region and/or white matter tract that was not clearly discernible or not present. Therefore, MLM was used to predict missing imaging values for indiscernible or non-present regions for a given participant. To predict these missing values, MLM uses the actual values and random effects of the other subjects in the model. This means that the predicted estimate is an average of other participants nested in the same group (FAST or placebo). The MLM estimated parameters were then imputed for the missing brain network connectivity metrics.

The MLM imputation procedures were validated by examining discrepancies between the observed metric and the estimated parameters using χ^2^ tests (see Supplement C for additional details on validation procedures). After these validation procedures, the MLM parameter estimates of missing brain connectivity metrics were used for all brain connectivity analyses.

### Data Used in Analyses: Indices of BL, EP, and Change

The MLM-derived neurobehavioral and brain connectivity measures, after verifications, were used in all data analyses. Each participant's predicted MLM parameters from each neurobehavioral and brain connectivity model were used to compute their BL (intercept) and EP [intercept + random intercept + (random slope + fixed slope) × time] neurobehavioral and brain connectivity measures. These computations of DOCS-25 Total, DOCS-25 Auditory-Language, CNC, rsFC, and FA were then used for all analyses as each patient's BL, EP, and change from BL to EP in neurobehavioral function and neural network connectivity.

To determine the influence of using imputed MLM parameters estimates for missing imaging metrics on revealing potentially important relationships, all analyses were repeated using the raw imaging metrics (i.e., see Tables SD1–3 in Supplement D for all raw/unmodeled *z*, FA values).

### Data Analyses: Examinations of Imaging Subgroup Representativeness

To identify potential sources of bias from the non-randomized selection of participants for this pilot study, analyses were conducted to examine the representativeness of the imaging subgroup to the RCT sample. Representativeness was examined by comparing (a) FAST RCT participants included in the imaging subgroup (*n* = 4) vs. FAST RCT participants excluded from the imaging subgroup (*n* = 4); and (b) placebo RCT participants included in the imaging subgroup (*n* = 4) vs. the excluded placebo RCT participants (*n* = 3).

The examinations of representativeness included comparisons of the MLM-predicted estimates of DOCS-25 Total, DOCS-25 Auditory-Language, and CNC measures of neurobehavioral function, as well as rsFC and DTI measures of brain connectivity. These examinations also compared injury severity, prognostic factors, demographics, and usual-care services. Differences in neurobehavioral measures were identified using 2-tailed Student *t*-tests. We also examined MLM derived trend lines.

### Data Analyses: Discovering and Identifying Robust Relationships

The process of discovering relationships and then identifying those that warrant further study involved significance testing. Significance testing was not used to establish the extent of the intervention effects. Considering the magnitude of the data and the goal of identifying relationships warranting further investigation, significance testing was used to filter the relationships revealed in discovery procedures, thereby identifying robust relationships. Significance testing was conducted (unless otherwise specified) using non-parametric permutation statistical testing as it is flexible and robust under model violations. This approach is also powerful for small samples in which verification of the distribution of the test statistic is unreliable (63). For permutation tests, no specific distribution of the test statistic under the null hypothesis is assumed. Test statistics were obtained by calculating all possible values of test statistics under rearrangements of the labels on the observed data points. The exact ordered *p*-values for the permutation tests were computed using the number of ordered statistics greater than the original test statistic, t1, and the total number of permuted test statistics. For example, when *n* = 4 subjects, and there are 3 ordered test statistics > t1, the test will have an ordered *p* = 3/2^4^ (i.e., = 3/16 where ordered *p* = 0.19). As multiple tests were involved with the significance testing (e.g., 10 tests for 10 within- and between-networks), we adjusted all ordered *p*-values by controlling the false discovery rate (FDR) using the Benjamini and Hochberg procedure with *q* = 0.20 (64) (see Figure SC3 in Supplement C for additional details and an illustration). We opted for a liberal FDR to avoid the possibility of missing important relationships that warrant further examination in future research.

Non-parametric significance testing procedures were also used to identify BL differences between FAST and placebo groups for each of the 26 gray matter ROIs and 19 white matter tracts. If there was a significant BL difference for any of the MLM derived brain connectivity measures, then this region or tract was not included in computation of correlations.

Pearson correlation coefficients were computed to reveal neural connectivity changes associated with neurobehavioral gains for the FAST and placebo interventions. For all brain connectivity measures not significantly different between FAST and placebo groups at BL, the MLM-derived rsFC and FA measures of change were used to compute correlations with MLM-derived DOCS-25 Total, DOCS-25 Auditory-Language and CNC measures of neurobehavioral change.

In the effort to identify robust relationships, significance of correlations between changes in neural connectivity and neurobehavioral gains as well as between resting state networks were tested. Significance was tested using *t*-tests for Pearson correlations and *t*-tests for Fisher *z* transformed values, respectively. We examined significance of correlations within and between FAST and placebo groups.

### Data Analyses: Verifying the Merits of a Positive Correlation for the FAST Intervention

When a significant positive correlation for FAST participants was identified using above procedures, then this relationship was verified by recomputing correlations using adjusted metrics of neural connectivity, specifically FA. The FA metrics, for specific fiber tracts, were adjusted by standardizing the amount of substrate (i.e., white fiber tract length) while controlling for individual brain size (i.e., normalization) (also see Supplement C for additional details on computations and verifications). Standardizing tract length, by using fixed length for a tract across subjects, is important as this accounts for injury heterogeneity. One subject may, for example, have substantially more intact fiber tract (i.e., longer discernible tracts in DTI) than another and, considering the small sample size, this could overestimate or underestimate the correlation with neurobehavioral gains. To avoid overestimation of the correlation, we examined the lengths of a given fiber tract across all subjects and then used the smallest length to define the tract length for all subjects. Using a fixed length across all subjects means that, for some subjects, partial volumes were used. However, the approach of using a fixed length, defined by the smallest length across subjects, neutralizes the possibility of overestimating the correlation. Notably, this approach could underestimate the correlation, but given the objective of verifying a positive correlation, we chose to avoid overestimation. Considering that each subject also has his/her own unique brain size, the fixed tract length was then normalized for each subject according to the normal persons' brain size (MNI template). In summary, for any fiber tract with changes in structural connectivity found to be significantly and positively correlated with neurobehavioral gains for FAST participants, we conducted a verification analysis. This verification involved adjusting the relevant FA measures to be (a) standardized by amount of substrate across subjects and (b) normalized by brain size according to MNI brain volume. To verify the finding of a positive correlation, we then re-estimated change in brain connectivity measures from BL to EP by using the adjusted FA measures in the MLM described above. These MLM re-estimated FA measures were then used to recompute Pearson correlations between FA change and neurobehavioral change. Correlations remaining positive were identified as robust relationships warranting further examination in future research.

## Results

Examinations of the precision of MLM estimated measures of brain connectivity metrics indicate very small MAE and MARE and no significant differences between the actual and estimated values (*p* = 0.999) (also see Table SC1 in Supplement C). Accordingly, all neural connectivity results are based on the MLM estimated z and FA measures. For reference, results based on raw neural connectivity values are provided in Tables SD4,5 in Supplement D.

### Representativeness of Pilot Study Participants

At BL, the RCT participants included in the imaging subgroup (*n* = 8) and the RCT participants excluded from the pilot study (*n* = 7) did not differ according to demographic factors (e.g., age, time post TBI), clinical states (e.g., VS, MCS), prognostic factors (e.g., comorbidities, time post-injury, lesion location, type), usual-care services (e.g., pharmacological, therapeutic content), or by DOCS-25-total and CNC measures of neurobehavioral function (all *p* > 0.05; see Table SE1 in Supplement E, columns A–C). The only difference between RCT participants excluded and included in the pilot study was cause of injury. The majority of excluded participants (5/7) and one included participant (1/8) were injured in automobile accidents (*p* = 0.01). The MLM-derived linear trend lines indicated that the RCT participants included in the pilot study were similar to the excluded participants according to neurobehavioral function (all *p* > 0.05).

### Pilot Study Participants: Imaging Subgroup Composition

The imaging subgroup (*n* = 8) comprised largely men (88%) who incurred a TBI at an average age of 40 years and who, at time of RCT enrollment, had remained in states of DoC an average of 78 days (Table 1, also see Supplement E). At BL, the majority (62%) of participants presented with behavioral characteristics consistent with MCS. At treatment EP, 25% of the subgroup remained in VS, 25% either progressed to or remained in MCS, and 50% emerged from MCS.

The pilot study participants who received the FAST (*n* = 4) vs. those receiving the placebo (*n* = 4) intervention did not differ at BL according to demographics, prognostic factors, and usual care (all *p* > 0.05; see Table SE1 in Supplement E, columns D–F). The groups also did not differ (*p* = 0.46) according to time between injury and study BL (FAST: mean = 117.0, SD = 56.2; median = 111, range = 59–187) (placebo: mean = 91.0, SD = 34.5; media*n* = 88; range = 52–136). The FAST and placebo participants also did not significantly differ by type and location of brain lesions, but placebo participants had a slightly higher number of contused brain regions and total number of lesions.

BL mean rsFC strength within each neural network was similar between FAST and placebo participants (Table 2, column G). For rsFC strength between-networks at BL, the FAST participants had significantly (ordered *p* = 0.04) weaker correlation (*z* = 0.62) between AN–DMN relative to participants receiving the placebo (*z* = 0.74).

**Table 2:** Resting state functional connectivity within and between networks: pilot study imaging subgroup FAST and placebo participants.

	**FAST (F*****_*******n*******_**=***** **4)**			**Placebo (PL*****_*******n*******_**=***** **4)**			**FAST vs. Placebo**	
	**A**	**B**	**C**	**D**	**E**	**F**	**G**	**H**
	**Mean correlations (*****Z*** values^*^)		**Change (EP—BL)**	**Mean correlations (*****Z*** values^*^)		**Change (EP—BL)**	**Baseline (BL)**	**Endpoint (EP)**
**Networks**	**Baseline**	**Endpoint**	**Ordered** ***p***	**Baseline**	**Endpoint**	**Ordered** ***p***	**Ordered** ***p***	**Ordered** ***p***
AN	0.32	0.29	**<0.01**	0.27	0.27	0.40	0.16	0.29
DMN	0.33	0.28	**0.06**	0.29	0.29	0.53	0.23	0.36
LN	0.52	0.41	**<0.01**	0.44	0.42	0.33	0.34	0.47
SN	0.47	0.30	**<0.01**	0.42	0.41	0.40	0.17	**0.04 (*****F*** **<** **PL)**
AN–DMN	0.62	0.67	0.53	0.74	0.67	**<0.01**	**0.04 (*****F*** **<** **PL)**	0.49
AN–LN	0.53	0.29	**<0.01**	0.44	0.53	**<0.01**	0.14	**0.0 (*****F*** **<** **PL)**
AN–SN	0.04	0.31	0.13	0.32	0.30	0.33	0.23	0.47
DMN–LN	0.67	0.65	0.13	0.54	0.56	0.53	0.13	0.34
DMN–SN	0.45	0.55	**<0.01**	0.47	0.43	0.27	0.55	0.27
LN–SN	0.47	0.76	0.13	0.74	0.68	0.27	**0.08**	0.35

* *z, mixed-effects linear model estimates; ordered p, independent-sample permuted t-tests conducted with mean estimated z values; Black shaded cells = ordered p ≤ 0.05 where significance testing accounted for multiple comparisons using FDR q = 0.20; Gray shaded cells = ordered p > 0.05 and < 0.10; F > or < PL: FAST group mean is > or < than placebo group mean at; AN, attention network; DMN, default mode network; FAST, familiar auditory sensory training; LN, language network; SN, salience network*.

BL FA values between the FAST and placebo participants (see Table SF5 in Supplement F) did not significantly differ for 18 of the 19 tracts examined (all *p* > 0.05). As the FA of the left inferior fronto-occipital fasciculus (IFOF) was significantly higher for the FAST participants (mean FA = 0.37, SD = 0.01; placebo mean FA = 0.35, SD = 0.01; *p* = 0.03), the FA for the left IFOF was not considered in final interpretations.

### FAST Facilitated Changes in Functional Connectivity

#### Within-Group Differences

The FAST participants had three significant within-network changes in rsFC strength. Specifically, mean *z* values decreased from BL to EP for the AN, LN, and SN (Table 2, column C, EP–BL). The FAST participants also had two significant between-network changes with rsFC strength: decreasing for AN-LN and increasing for DMN-SN (Table 2, column C).

There were no significant within-network rsFC strength changes for the placebo participants (Table 2, column F). There were, however, significant rsFC changes between-networks: rsFC of AN–DMN decreased and AN-LN increased.

#### Between-Group Differences

At treatment EP, the placebo participants had significantly stronger rsFC within SN. The mean *z* value for the FAST group declined from 0.47 to 0.30 but remained stable for the placebo group (0.42 and 0.41). The placebo participants also had significantly stronger rsFC between AN-LN (Table 2, column H) with mean *z* for AN-LN increasing for the placebo group and decreasing for the FAST group.

### FAST Facilitated Changes in Structural Connectivity

#### Within-Group Differences

**Figure 2:**
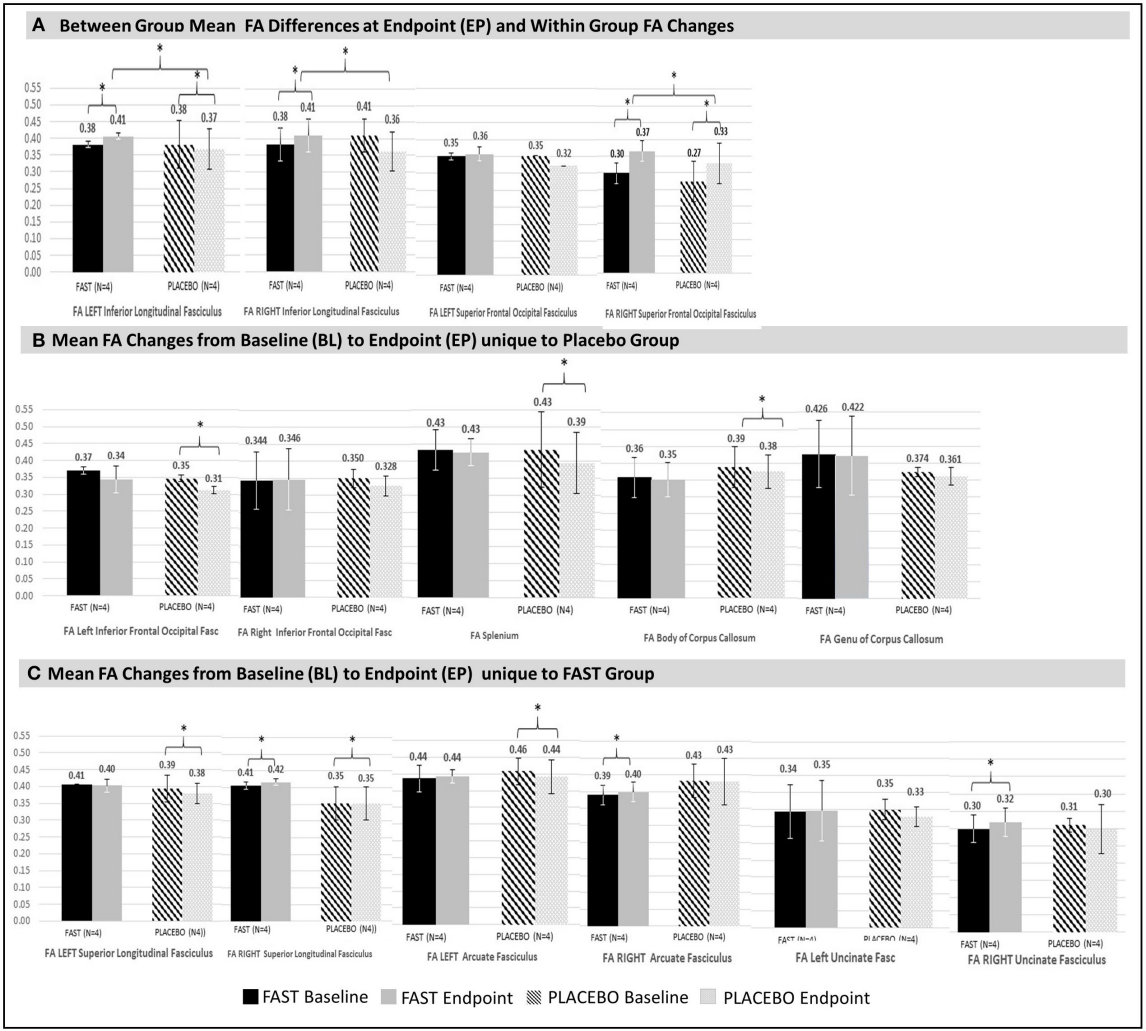
Significant changes in structural connectivity between and within groups. Significant (denoted with * with all *p* < 0.00) mean FA differences between and within groups at endpoint are illustrated. For comparison purposes, non-significant FA values for contralateral tracts are also provided. Vertical bars in black and white indicate standard deviations. FAST group is denoted with solid black (baseline) and gray (endpoint) bars. Placebo group is denoted with patterned black (baseline) and patterned gray (endpoint) bars. **(A)** Between- and within-groups: Between-group mean FA endpoint differences and within-group FA changes from baseline to endpoint. FAST group's FAs for the left and right ILF at endpoint are greater than same measures for Placebo group. Change in FA of the right SFOF increases for both groups, but the FAST group increases more than the placebo group. **(B)** Placebo group: Mean FA changes from baseline to endpoint within tracts unique to the placebo group were all decreases. Decreases occurred in the right AF, splenium, and body of corpus callosum. The left IFOF also significantly declined, but at baseline this was significantly lower for the placebo group. Thus, the left IFOF finding is not considered for interpretation. Although there were no significant changes for the genu, this tract is shown to facilitate evaluation of all colossal fiber tracts. **(C)** FAST group: Mean FA changes from baseline to endpoint that are unique for the FAST group occurred within three right hemisphere tracts: right SLF, the right arcuate fasciculus (AF), and the right uncinate fasciculus (UF).

FAST participants had significant increases in unadjusted FA for three tracts: the right superior longitudinal fasciculus (SLF), the right arcuate fasciculus (AF), and the right uncinate fasciculus (UF) (Figure 2C; also see Table SF1 in Supplement F). All three tracts decreased bilaterally for the placebo participants, with decreases in the right AF reaching statistical significance.

The placebo participants had significant FA declines of the splenium and body of the corpus callosum, whereas FAST participants had non-significant declines in FA for these same tracts (Figure 2B).

#### Between-Group Differences

There were significant differences between FAST and placebo participants in the unadjusted FA at EP, for the left and right inferior longitudinal fasciculus (ILF) (Figure 2A). This finding was due to a decrease within the ILF for the placebo participants and an increase for the FAST participants. Both groups demonstrated an increase of the unadjusted FA of the right superior frontal-occipital fasciculus (SFOF); the FAST participants increased more than the placebo participants.

### Relationships Between Changes in Connectivity and Neurobehavioral Gains

The MLM-derived measures of change in neurobehavioral function and neural connectivity (rsFC and DTI) were used to reveal FAST treatment-related changes in neural connectivity associated with neurobehavioral recovery. For rsFC, there were no significant associations between indices of neurobehavioral change within any rsFC network or between any network–network pair (see Tables SF2, 3 in Supplement F). For DTI, however, the FAST participants' changes in mean FA of the left ILF were significantly and positively correlated with the DOCS-25 Auditory-Language measure (*p* = 0.02). For placebo participants, increased FA of right UF was also significantly and positively correlated to improving arousal and awareness as measured by the CNC (*p* = 0.03) (also see Figure SF1 in Supplement F).

### Verifying the Relationship Between ILF Change and FAST-Related Neurobehavioral Gains

**Figure 3:**
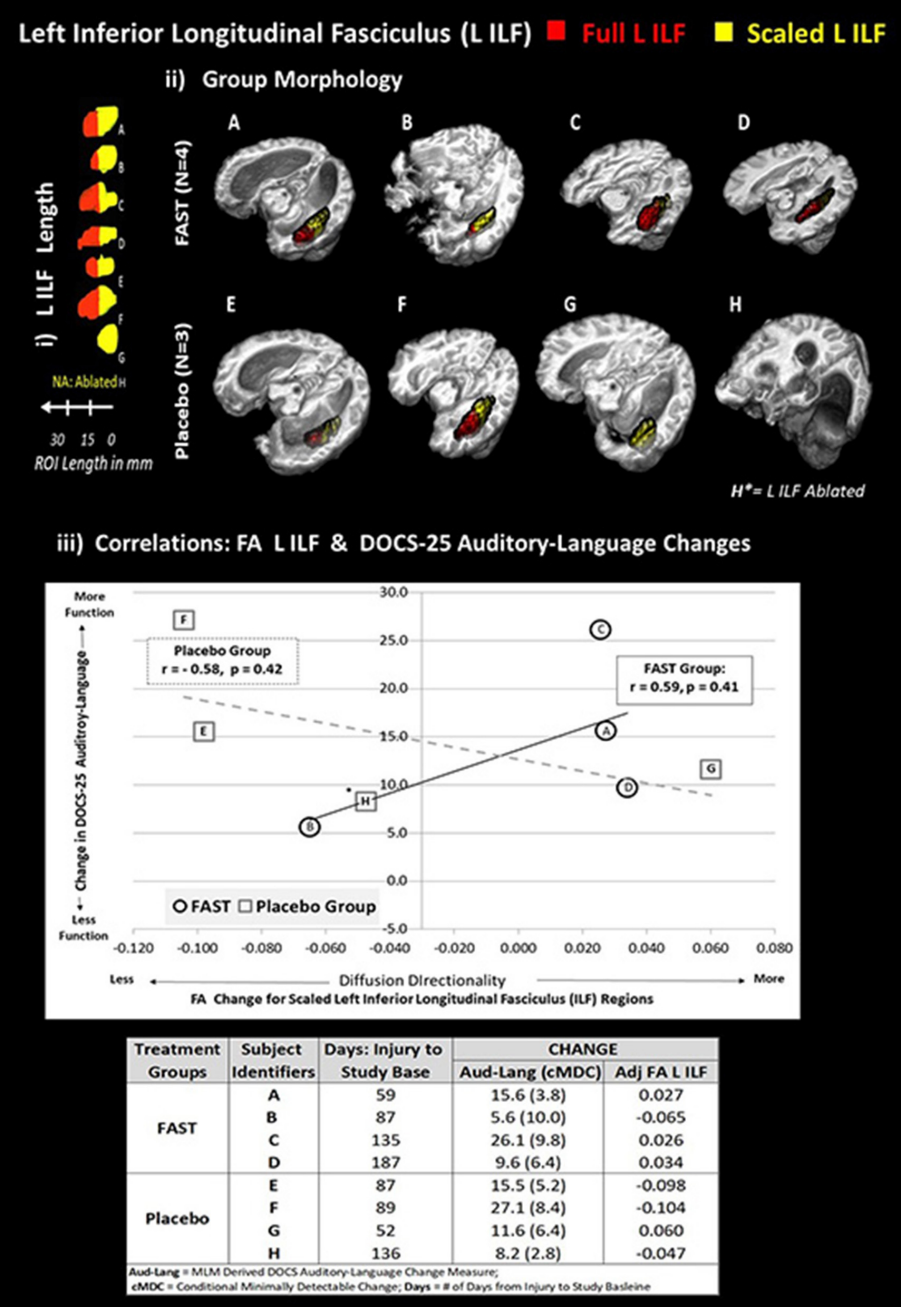
Each subject's left ILF by group and correlation with DOCS-25 auditory-language measures. The top half of figure illustrates the full left ILF, depicted in red and adjusted left ILF, depicted in yellow. Panel i shows the length in millimeters (mm). Panel ii demonstrates the group-level morphology in native space. To standardize the amount of substrate, relative brain size, and location of the ILF comparisons, an adjusted portion of each subject's left ILF was used to compare FA values. The following formula was applied: Adjusted left ILF = [(subject's skull length a–p)/(MNI skull length a–p) ^*^ shortest ILF].
All ILFs were limited to shortest ILF in the in anterior to posterior (a–p) plane (i.e., G had the shortest ILF at 14 voxels with 1-mm^3^ resolution).The 14-voxel length was then scaled for each subject based on the ratio of their maximum skull length over the maximum skull length of the MNI152 brain (both in the a–p plane).To extract comparable FA values, the adjusted left ILF length was measured and masked from the posterior pole of the ILF in the a–p plane. Plot on bottom half of figure panel iii indicates that the scaled FA left ILF values are, for the FAST group, positively correlated with improved DOCS-25 Auditory-Language abilities. The plot depicts trend lines by group for change between baseline and endpoint in scaled left ILF FA and DOCS-25 Auditory-Language measures reported for each subject. ^*^Subject H is missing the left ILF, and this FA value was estimated using imputation methods. Each subject's alphabetic label corresponds with panels i and ii and the change indices provided in the table in bottom of panel iii.

The positive correlation for FAST participants between the left ILF and the DOCS Auditory-Language gains was verified by recomputing Pearson correlations using the adjusted FA for the left ILF (Figure 3, i and ii). Although not significant (*p* > 0.05), the correlation using the adjusted FA measures remained positive and moderately strong (*r* = 0.59) for FAST participants (Figure 3, iii). For the placebo participants, however, the correlations changed from positive to negative (*r* = −0.58).

As depicted in Figure 3 (panel iii and Data table), three of the four FAST participants and all four of the placebo participants made meaningful DOCS-25 Auditory-Language gains. Specifically, meaningful gains are indicated when the participant's neurobehavioral gains exceed their measurement error defined as the conditional minimally detectable change (65) Figure 3 also indicates that the adjusted FA measures for the left ILF improved for the same three FAST participants (A, C, and D), but decreased for three of the four placebo participants. More specifically, these three FAST participants made gains in the adjusted FA of the left ILF and the DOCS Auditory-Language measures. Participant A was earliest after injury (59 days) and made more gains than the other two participants C and D who were studied 135 and 187 days post-injury, respectively. In contrast, the only placebo participant making gains in both DOCS auditory-language skills and the adjusted FA of left ILF was participant G, who, of all the placebo participants, was studied the earliest after injury (52 days).

## Discussion

In our pilot study of persons with DoC due to TBI, we examined longitudinal data for an imaging subgroup derived from a double-blind placebo-controlled RCT (33). We discovered relationships between the FAST intervention and changes in structural and functional neural connectivity, one of which was positively associated with neurobehavioral gains. To identify FAST-related relationships warranting further examination in future research, we used significance testing to filter the revealed relationships, and we verified the positive correlation. For a patient population with many scientific challenges, this report demonstrates a systematic approach to rigorously explicating these relationships. This scientific approach provides a basis for identifying the unique contributions of specific interventions to recovery, thereby providing a foundation for developing complementary treatments for a patient population with limited treatment options. If the FAST intervention is examined further, and findings are replicated in future research, then these findings could delineate the unique contributions of the FAST intervention to recovery and, ultimately, inform development of targeted treatment strategies to improve brain and behavioral function for persons with DoC after TBI.

### FAST Intervention: Structural Connectivity and Auditory-Language Skills

A key finding that merits replication in future research is the positive association between the FA of the left ILF and the DOCS-25 Auditory-Language measures. Further investigation is merited, in part, because the positive direction of the association for the FAST group was verified by recomputing the correlations using an adjusted FA. Considering that adjusting the FA involved using, across all subjects, the smallest length of the left ILF to define a fixed tract length for all subjects, this approach means that we used partial volumes for the other participants, thereby underestimating the strength of the correlation. As the objective was to verify the presence of a robust relationship, this approach was selected because it avoided overestimation. Additional evidence indicating that this relationship is robust includes the findings that the FAST group had significantly higher mean FA of the left IL at treatment EP (Figure 2A). Furthermore, findings suggest that time post-injury or the potential for change in structural connectivity due to innate recovery did not prohibit gains in the FA of the left ILF. Descriptive findings indicate that the time post-injury was balanced between the FAST and placebo groups. Also, three of the four FAST participants and only one placebo participant made gains in both the auditory-language skills and the FA of Left ILF. Notably, this placebo participant was the earliest after injury, whereas the FAST participants ranged from 59 to 187 days after injury. Considering that (a) both the FAST and placebo groups improved in DOCS-25 auditory-language skills, (b) the adjusted FA of the left ILF increased for the majority of the FAST participants but decreased for the majority of the placebo participants, and (c) the collective evidence suggests that time post-injury did not prohibit gains in FA of the left ILF, future research should focus on understanding the relationship between the FAST and left ILF, particularly the role of the left ILF relative to time post-injury.

We speculate that additional future examinations could indicate that the FAST intervention facilitated connectivity changes and/or prevented degradation within the left ILF. Based on our RCT findings (33) and a previous case report, (66) we also speculate that these findings will be present in the acute through the chronic stages of recovery but that the effects will be more pronounced earlier after injury.

Future research to further advance the understanding of likelihood of these FAST intervention effects and/or explicating other effects of the FAST within the left ILF is particularly valuable when considering the involvement of the left ILF in language function. The left ILF is a long intrahemispheric association pathway connecting the anterior temporal lobe to the occipital lobe (67, 68) intersecting with the posterior segments of the AF, the UF, and the IFOF. Importantly, these three tracts are all thought to serve as components of the ventral language pathway, and each is implicated in the rehearsal component of language recovery (69, 70). The IFOF is also thought to play a particularly important role in semantic processing. Because the unadjusted FA of the Left IFOF was significantly higher for the FAST participants at BL, we did not examine its role. Future studies should, however, examine the role of the left IFOF relative to the left ILF. Further consideration of (i) the role of the ventral language pathway in providing repeated and recurring opportunities for learning, particularly when there are constraints on working memory, (71) and that (ii) the FAST intervention provides repeated exposure to language-based episodic stories also highlight the need to identify the unique and/or synergistic contributions of the features of the autobiographical auditory-linguistic stimuli (i.e., language, speech patterns, autobiographical content, emotions, familiar voices). More specifically, the FAST RCT was not designed to delineate the roles and contributions of each feature alone vs. together or to explicate the amount of repetition necessary (i.e., dose).

A final consideration regarding the merits of future examinations of this complex relationship is the potential impact of replicating an association between the left ILF and DOCS-25 auditory-language skills. If replicated, then this association would indicate that the FAST intervention, consisting of autobiographical auditory-linguistic stimuli, provides targeted stimulation to specific neural networks resulting in alterations of structural connectivity, and importantly, further explication would advance understanding of the role of these changes in supporting auditory-language skills. Given the importance of the left ILF to language and recovery of consciousness, (72) our findings of increased left ILF connectivity with provision of the FAST intervention as well as the potential clinical impact, the presence and nature of the complex relationship among the FAST intervention, the left LF and DOCS-25 auditory-language skills merits additional examination in future research.

### FAST Intervention: Structural Connectivity of Right Hemisphere Language Homologs

An additional relationship identified as meriting further investigation is the finding that only the FAST group had increased structural connectivity of the right SLF and right AF without any neurobehavioral associations. If this finding is replicated in future research, we theorize that this would indicate that the FAST intervention facilitates redirection of resources to right hemisphere language homologs and that these changes may not be sufficient to support neurobehavioral recovery, and/or these changes precede neurobehavioral recovery. This hypothesis is plausible when considering that the right SLF, right AF, and left ILF are long-range association fiber tracts with overlapping cortical projections supporting multiple complex functions via polymodal brain hubs (68, 73–78). Although there is evidence that DoC patients have an impaired ability for a brain hub to connect with spatially distant hubs, (79) these long-range association fiber tracts are capable of responding to multiple modalities. Also congruent with this hypothesis is prior research showing that function within these polymodal areas recovers after patients regain conscious behaviors (80). The idea of FAST treatment–induced resource redistribution is similar to previous suggestions of resource distribution related to the provision of usual care (81). The plausibility of FAST-induced redistribution is bolstered by a growing body of evidence indicating that the right hemisphere plays important roles in facilitating language functions and recovery after brain injury (33, 69, 82–84) and by our previously reported findings that FAST RCT participants had increased neural activation in the right hemisphere homologs of language processing in response to a non-familiar voice reading aloud a novel story (33).

We considered the possibility that, rather than redistribution, the FAST intervention engaged the right SLF and AF in a manner similar to the relateralization of language processing to intact homotopic right-hemisphere regions observed in language recovery after left hemisphere stroke. (27, 28, 85–87). However, the literature regarding right hemisphere compensatory functions is mixed with some findings, suggesting that right hemisphere engagement is pathological (88–91). When considering the findings of increased connectivity of the right SLF and AF in the context of increased FA in the left ILF, we posit that the injured brain's response to the autobiographical auditory-linguistic stimuli could include engagement of targeted networks that serve to redirect brain resources rather than a clear relateralization effect seen in language recovery with aphasia after stroke (83). The absence of correlations between the FA increases in right SLF and AF and neurobehavioral gains highlights the need for future mechanistic research investigating the idea of redistribution as well as to determine the necessary and sufficient levels of connectivity within the right SLF and AF to support neurobehavioral gains.

### Structural Connectivity Changes Relative to Usual Care and Endogenous Recovery

Both FAST and placebo groups had significant increases in FA of the right SFOF from BL to EP. Because both groups received usual care, this finding suggests that usual care and/or endogenous recovery contributed to the increases in structural connectivity of this fiber tract. Notably, the FA increased more for the FAST group such that the FA was significantly higher at EP compared to the placebo group (Figure 2A). Replication of this finding in future research would suggest that usual care and/or endogenous recovery engage the right SFOF and that there is more engagement when the FAST intervention is paired with usual care.

### Functional Connectivity Changes for FAST and Placebo

For the FAST group, rsFC decreased within the AN, LN, and the SN and increased between the SN and DMN (Table 2). Given the roles of each network and that the FAST stimuli are intended to target these networks, rsFC changes within and between these networks were expected. The direction of several of the rsFC changes, however, was unexpected. The rsFC of the AN-LN, for example, decreased in the FAST group and increased for the placebo group (Table 2). We expected the opposite, in part, because the linguistic components of the FAST stories are designed with the intention of engaging the LN (69). Furthermore, the AN supports the ability to volitionally (i.e., dorsal AN) attend to a task and to reflexively (i.e., ventral AN) attend to or detect a stimulus (92–94). These unexpected findings, in terms of direction of changes, are noteworthy because the degree of hyperconnectivity and hypoconnectivity in DoC remains poorly understood (8, 22–26) and warrants further research to inform therapeutic strategies.

Given the features of the stimuli used in the FAST intervention and the roles of each rsFC network, we were also surprised that changes in rsFC in the FAST group were not correlated to neurobehavioral recovery. The SN, for example, is thought to be targeted with the personalized auditory-linguistic stimuli (37) because this network supports the ability to orient toward salient emotional stimuli, (95) consciously perceive stimuli, (96–100) and detect and attend to stimuli (92–94). After TBI, coordinated SN–DMN interaction is also impaired, (101) and the discordance of interactions impedes efficient attention switching from internal processes to salient external stimuli. The surprising lack of associations may be related to the small sample size, which also precluded an examination of paired regions.

In lieu of interpreting these unexpected findings based on a small sample, we highlight them here to note the need to reexamine associations between rsFC changes and neurobehavioral recovery with a larger sample. The relationship between the FAST and changes in rsFC of these networks is an important area for further research, in part, because of knowledge that overt participation in a task requiring focused attention is necessary to activate the AN, LN, and SN. Thus, our findings of FAST intervention–related rsFC changes represent the first reported evidence that these broad neural networks can be engaged with a sensory treatment that does not require overt task participation (102–104). If future research findings are consistent, then clinicians will be able to provide treatments targeting broad neural networks important to recovery.

### Limitations

The robust methods employed in this pilot study, combined with use of data from a double-blind placebo-controlled RCT, provide confidence in the reported findings; however, the small sample size places limits on generalizability to the larger DoC population. The verified association between the adjusted FA of the left ILF and DOCS-25 Auditory-Language measures, for example, is based on four subjects per group. While this is a potentially important finding, it should be replicated in future research. The purpose of the study was to identify relationships meriting further investigation, and accordingly, we used FDR threshold of 0.20 for multiple comparisons. Although we chose this liberal threshold to avoid obscuring relationships that could provide insights for future research, this threshold means that for every 5 discoveries, there would be one false discovery and four true discoveries (105).

The imaging data, particularly DTI, were collected prior to the development of methods to correct for geometrical distortions (e.g., field maps and reversed phase encode). We did, however, correct for geometric distortions in our diffusion processing pipeline. First, the b-0 image was non-linearly transformed to the 3D anatomic scan. Next, each gradient direction was individually transformed to the *b* = 0 image through non-linear warping. This approach matches the diffusion data to the anatomic data reasonably well. Furthermore, the results are not in regions that might exhibit higher levels of distortion such as the orbital frontal cortex or brainstem.

Within-subject variability unique to rsFC data may not have been sufficiently minimized (106–109). The uniqueness of the within-subject variability associated with rsFC together with the small sample size for between-group comparisons indicates a need for replication of the rsFC findings or implementation of a different design that allows for examination of associations with neurobehavioral changes. The rsFC analyses and findings provide a framework that can be applied when replicating this study.

All analyses were conducted using a framework of *a priori* selected networks. While this approach is critical to advancing knowledge in unchartered areas of science, it also means that some changes in connectivity may have gone undetected (110). The *a priori* approach also limits the ability to identify connectivity between-network nodes (e.g., right SLF) with other polymodal hubs, which in turn engage other networks.

For our *a priori* approach with rsFC, we used established resting state networks consisting of gray matter regions with established roles. This approach allows us to interpret our findings relative to other studies, but this also means that the auditory-language network is a particularly simplistic representation of the language connectome (69, 111). For the unadjusted FA values, we also drew full white matter tracts for each subject because we sought to explore the function of the entire tract. This approach of averaging the entire white fiber tract assumes equipotentiality within each white matter tract. As a result, we may not have detected more subtle changes in structural connectivity.

### Future Directions

Future research examining the relationships identified above as well as potential connections between the left ILF, right AF, and right SLF with other networks can provide insights about how to target treatments to support recovery of auditory-language skills. Determining the unique contributions of any treatment to neurobehavioral recovery requires the development of methods to address the many challenges with DoC research. Thus, the methods employed in our scientific approach must be replicated and refined to identify the best approach for modeling these complex data. Future research also needs to systematically define clinically meaningful changes in functional and structural connectivity for people with DoC that can be used to examine efficacy of targeted treatments in clinical trials. The replication and further examination of these important relationships, refinement of the methods we employed, and advancing knowledge of clinically meaningful changes in neural connectivity will further enable determination of the unique contributions of the FAST and other interventions and inform development of targeted treatments.

The state of DoC science informed our network-level approach to identify functional and structural connectivity changes and to examine the relationship between these changes and neurobehavioral gains. This method, though, is not a direct causal approach and precludes definitive determinations of mechanisms. Further research examining the role or contributions of alternate white matter pathways to neurobehavioral recovery from DoC after TBI is needed to definitively identify viable therapeutic targets.

Prior research has demonstrated that language gains are likely to be related to white matter microstructure and association fibers that link gray matter regions to enable the cross-modal integration required for higher-order complex behaviors (112). Consistent with this premise is the reported finding of no correlations between rsFC and neurobehavioral changes and presence of positive correlation between increasing structural connectivity and neurobehavioral gains. This consistency between previous research and our findings identifies a need for research examining the convergence and divergence of rsFC and structural connectivity during neurobehavioral recovery using a direct causal approach (113).

To develop effective neuromodulatory interventions, studies similar to ongoing research in stroke (21) are needed to advance knowledge of the relationship of hyperconnectivity and hypoconnectivity with neurobehavioral recovery from DoC. The unexpected findings of direction of rsFC changes highlight the need for research determining the degree that hyperconnectivity and hypoconnectivity in DoC is pathological (8, 22–26). Investigating this construct is important for informing development of treatments targeting attenuation or excitation of functional connections.

The incongruences in some of the reported findings highlight the challenges involved in characterizing and interpreting recovery and/or reconfiguration of network connectivity in a severely damaged system, particularly when considering novel patterns of activation/deactivation related to a specific treatment. The incongruences highlight the importance of considering how a damaged network may fluctuate over time. It is plausible that treatment-induced connectivity changes cause a cascade of fluctuations in activations and deactivations that unfold over different time scales as each network attempts to repair itself. We have addressed this challenge by using longitudinal data derived from a placebo-controlled RCT, thereby enabling a comparison of the FAST and placebo groups. Future work, however, would benefit from taking repeated measures at more than two timepoints during provision of the intervention to assess within-subject variability. This challenge highlights the need for future research on how the damaged brain repairs and recovers as well as how treatment alters this recovery. Higher-order diffusion models that can account for multiple fibers in a single voxel are also needed to advance this knowledge.

## Conclusions

For persons with DoC after TBI, our findings collectively represent an important first step toward understanding the unique contributions of the FAST intervention, which is a simple passive sensory treatment consisting of autobiographical auditory-linguistic stimuli that are provided with the intention of engaging specific and broad neural networks known to be important to recovery. The findings warranting further investigation and replication in future research are those suggesting that the FAST may induce structural and functional connectivity changes, some of which associated with auditory-language gains.

The reported findings make unique and clinically meaningful contributions to the field of neurorehabilitation while expanding on previously published findings demonstrating that the FAST intervention is related to improved awareness and language skills. While there are reports of increased task activation after recovery to passive sensory stimulation in differing gray matter regions, (33, 81, 114) to our knowledge there has never been a placebo-controlled RCT-based report of resting state functional and structural connectivity changes related to provision of a passive yet targeted sensory stimulation treatment in DoC patients. Generalizability of our findings to all patients with DoC is, nonetheless, premature. If our findings are supported in future studies, however, then this low-cost, effective treatment can be readily modified and clinically implemented. For a patient population with limited treatment options, this line of research could have important therapeutic implications.

## Data Availability Statement

All datasets generated for this study are included in the article/Supplementary Material.

## Ethics Statement

The studies involving human participants were reviewed and approved by Hines VA, Richmond VA and Northwestern Unviersity's IRBs. The patients/participants provided their written informed consent to participate in this study.

## Author Contributions

All authors made substantial contributions to the conception or design of the work, or the acquisition, analysis or interpretation of data for the work, and drafted the submitted work and/or revised it critically for important intellectual content, and also provide approval for publication of the content and agree to be accountable for all aspects of the work in ensuring that questions related to the accuracy or integrity of any part of the work are appropriately investigated and resolved.

## Conflict of Interest

The authors declare that the research was conducted in the absence of any commercial or financial relationships that could be construed as a potential conflict of interest.

## Abbreviations

AF: arcuate fasciculus
AN: attention network
BL: baseline
BOLD: blood oxygen level–dependent
CNC Scale: Coma–Near-Coma Scale
DARTEL: Diffeomorphic Anatomical Registration Through Exponentiated Lie algebra; a suite of tools for more accurate intersubject registration of brain images
DMN: default mode network
DoC: disordered consciousness
DOCS-25: Disorders of Consciousness Scale-25 (2014 version with 25 calibrated items)
DTI: diffusion tensor imaging
DVARS: D = temporal derivative of the time course; VARS = root mean square of the variance over voxels framewise displacement relative to the root mean square signal change; spatial standard deviation of successive difference image
eMCS: emergence from minimally conscious state
EP: endpoint
EPI: echo planar images
F: FAST (F) group
FA: fractional anisotropy
FAST: familiar auditory sensory training
FD: framewise displacement
FDR: false discovery rate
FOV: field of view
FSL: FMRI Software Library
IFOF: left inferior fronto-occipital fasciculus
ILF: inferior longitudinal fasciculus
LN: language network
MCS: minimally conscious state
MFRM: multi-faceted Rasch measurement
MLM: mixed linear effects models
MNI152: Montreal Neurological Institute 152 template
Ordered p: independent sample permuted *t*-tests conducted with mean estimated *z* values
RCT: randomized clinical trial
ROI: region of interest
rsFC: resting state functional connectivity
rVLPFC: right ventral lateral prefrontal cortex
SD: standard deviation
SFOF: superior frontal-occipital fasciculus
SLF: superior longitudinal fasciculus
SN: salience network
TBI: traumatic brain injury
TE: echo time
TR: repetition time
UF: uncinate fasciculus
VS: vegetative state
*Z*: mixed-effects linear model estimates.
