# Supplementary material for: Neural Connectivity Changes Facilitated by Familiar Auditory Sensory Training in Disordered Consciousness: A TBI Pilot Study
Source: Front Neurol. 2020 Oct 8;11:1027. doi: 10.3389/fneur.2020.01027 (PMC7578344; doi:10.3389/fneur.2020.01027)
Supplement: Supplementary file 1 [file Data_Sheet_1.DOCX]

**Supplement A: Elaboration on Details of FAST Intervention**

The FAST RCT, as previously reported,^1^ investigated the efficacy of the FAST intervention (FAST group) in contrast to a placebo intervention (i.e., a blank recording (silence) of the same frequency and duration as the FAST recordings). The FAST intervention used in the RCT has been previously described according to theoretical framework and operational procedures.^2^ Here we provide additional unpublished details and/or elaborate on previously published descriptions.

The FAST intervention involved repeated provision of personalized auditory-linguistic stimuli based on five familiarity features: (1) language, (2) speech patterns, (3) autobiographical content, (4) emotions, and (5) familiar voices. These features were embedded in audio recordings of language-based stories about events familiar to the patient as recorded by the person who participated in the event with the patient (typically a close family member). As reported elsewhere, the FAST intervention ^2^ is based on the premise that repeated provision of auditory linguistic-stimuli of familiar voices telling stories about mutually-experienced events and intrinsic emotions, will provide sufficient intensity to modulate broad neural networks. More specifically, repeated provision of the FAST intervention is intended to evoke content-rich autobiographical memory traces that, in turn, modulate broad neural networks.

For the RCT, the stimuli used with the FAST was standardized across subjects according to (a) story-teller eligibility, (b) autobiographical-episodic content, (c) balanced affective valence, (d) total amount of unique story content, and (e) story delivery specifications. To create the autobiographical auditory-linguistic stimuli, 40 minutes of stories were recorded and divided into four 10-minute segments. To ensure that all five familiarity features were included in each story, the duration of each story was allowed to vary across subjects. Each of the four 10-minute segments, however, included at least two unique stories and each segment was balanced for affective valence with approximately 3.3 minutes allocated to each of three emotional elements (sad, happy and neutral). The four 10-minute segments were each played once per day, in randomized order, for six weeks. For the RCT, all stories were in English as participants were only eligible if they were verbally fluent in English.

Previously published RCT results^1^ indicate that, as expected, both the FAST and Placebo groups improved clinically, but the FAST group had significantly more neurobehavioral gains on the Coma-Near-Coma (CNC) scale and increased activation in response to both familiar and non-familiar vocal stimuli during task-based functional MRI (fMRI). Although the RCT was not designed to identify which of the five familiarity features embedded in each FAST story actively modulated the broad neural networks, RCT findings indicated that the FAST treatment induced increased neural activation in right hemisphere language homologues, suggesting network-level reorganization similar to compensatory mechanisms reported for language recovery after stroke.^3,4^

The results of the RCT suggested that there may be changes in broad-scale neural networks induced by the FAST intervention. To examine this possibility, we conducted the present post-hoc study using a network-level approach focusing on the broad networks presumed to be targeted with the autobiographical auditory-linguistic stimuli used in the FAST intervention that was examined in the RCT:^2^ the language network (LN), salience network (SN), attention network (AN), and default mode network (DMN) (Figure 1). The linguistic components of the stimuli were selected based on research showing associations between increased rsFC within regions comprising the LN (e.g., Heschl’s) ^5,6^ and more consciousness.^7,8^ Familiar episodic stories re-told by a familiar person were used because the SN supports the ability to orient toward salient emotional stimuli,^9^ consciously perceive stimuli,^10-14^ and facilitate switching from internal to external processing.^15-17^ In addition, the AN supports the ability to attend to a stimulus either volitionally (i.e., dorsal AN) or reflexively (i.e., Ventral AN).^18-20^ The strength of rsFC within the SN is also thought to differentiate MCS from VS and rsFC strength of the AN during a volitional attention task is associated with degree of consciousness.^8,21^ While DTI studies of recovery from DoC are limited, structural connectivity between nodes of the SN (i.e., anterior cingulate cortex-ACC, amygdala) and external structures (i.e., prefrontal cortex-PFC) are also positively correlated with states of DoC in which more white matter connectivity is associated with behaviors indicative of more clinical consciousness.^22^

The DMN is closely associated with the function of the LN, SN and AN. Functionally, the health of the DMN reflects the ability of the CNS to switch between internal (e.g., mind wandering) and external processing (e.g., in response to external stimuli),^23-27^ a neural function speculated to be central to the emergence of consciousness and neurobehavioral recovery in DoC. Lower rsFC of the DMN, for example, is associated with less consciousness.^8,23,26,28-33^ Conversely, multiple reports indicate that stronger rsFC within and between DMN nodes (e.g., Posterior Cingulate Cortex (PCC); PCC and medial prefrontal cortex (PFC)) is associated with better neurobehavioral recovery.^32-36^ Furthermore, signs of emerging consciousness are positively associated with connectivity of white fiber tracts underlying the DMN, specifically in the posterior regions (i.e., PCC and Temporal Parietal Junction (TPJ)).^37,38^

1. Pape TL, Rosenow JM, Steiner M, et al. Placebo-Controlled Trial of Familiar Auditory Sensory Training for Acute Severe Traumatic Brain Injury: A Preliminary Report. *Neurorehabilitation and neural repair.* 2015;29(6):537-547.

2. Pape T, Rosenow J, Harton B, et al. Preliminary framework for a Familiar Auditory Sensory Training Task (FAST) provided during Coma Recovery. *Journal of Rehabilitation Research and Development* 2012;49(7):1137-1152.

3. Calvert GA, Brammer MJ, Morris RG, Williams SC, King N, Matthews PM. Using fMRI to study recovery from acquired dysphasia. *Brain and language.* 2000;71(3):391-399.

4. Rijntjes M, Weiller C. Recovery of motor and language abilities after stroke: the contribution of functional imaging. *Progress in neurobiology.* 2002;66(2):109-122.

5. Geschwind N. Disconnexion syndromes in animals and man (part 1). *Brain : a journal of neurology.* 1965;88(2):237-294.

6. Geschwind N. Disconnexion syndromes in animals and man (part 2). *Brain : a journal of neurology.* 1965;88(3):585-644.

7. Bruno MA, Majerus S, Boly M, et al. Functional neuroanatomy underlying the clinical subcategorization of minimally conscious state patients. *Journal of neurology.* 2012;259(6):1087-1098.

8. Demertzi A, Antonopoulos G, Heine L, et al. Intrinsic functional connectivity differentiates minimally conscious from unresponsive patients. *Brain : a journal of neurology.* 2015;138(Pt 9):2619-2631.

9. Seeley WW, Menon V, Schatzberg AF, et al. Dissociable intrinsic connectivity networks for salience processing and executive control. *The Journal of neuroscience : the official journal of the Society for Neuroscience.* 2007;27(9):2349-2356.

10. Boly M, Coleman MR, Davis MH, et al. When thoughts become action: an fMRI paradigm to study volitional brain activity in non-communicative brain injured patients. *NeuroImage.* 2007;36(3):979-992.

11. Langsjo JW, Alkire MT, Kaskinoro K, et al. Returning from oblivion: imaging the neural core of consciousness. *The Journal of neuroscience : the official journal of the Society for Neuroscience.* 2012;32(14):4935-4943.

12. Crone JS, Holler Y, Bergmann J, Golaszewski S, Trinka E, Kronbichler M. Self-related processing and deactivation of cortical midline regions in disorders of consciousness. *Frontiers in human neuroscience.* 2013;7:504.

13. Medford N, Critchley HD. Conjoint activity of anterior insular and anterior cingulate cortex: awareness and response. *Brain structure & function.* 2010;214(5-6):535-549.

14. Sadaghiani S, Scheeringa R, Lehongre K, Morillon B, Giraud AL, Kleinschmidt A. Intrinsic connectivity networks, alpha oscillations, and tonic alertness: a simultaneous electroencephalography/functional magnetic resonance imaging study. *The Journal of neuroscience : the official journal of the Society for Neuroscience.* 2010;30(30):10243-10250.

15. Menon V, Uddin LQ. Saliency, switching, attention and control: a network model of insula function. *Brain structure & function.* 2010;214(5-6):655-667.

16. Sridharan D, Levitin DJ, Menon V. A critical role for the right fronto-insular cortex in switching between central-executive and default-mode networks. *Proceedings of the National Academy of Sciences of the United States of America.* 2008;105(34):12569-12574.

17. Bonnelle V, Ham TE, Leech R, et al. Salience network integrity predicts default mode network function after traumatic brain injury. *Proceedings of the National Academy of Sciences of the United States of America.* 2012;109(12):4690-4695.

18. Corbetta M, Kincade JM, Ollinger JM, McAvoy MP, Shulman GL. Voluntary orienting is dissociated from target detection in human posterior parietal cortex. *Nature neuroscience.* 2000;3(3):292-297.

19. Corbetta M, Patel G, Shulman GL. The reorienting system of the human brain: from environment to theory of mind. *Neuron.* 2008;58(3):306-324.

20. Corbetta M, Shulman GL. Control of goal-directed and stimulus-driven attention in the brain. *Nature reviews Neuroscience.* 2002;3(3):201-215.

21. Qin P, Wu X, Huang Z, et al. How are different neural networks related to consciousness? *Annals of neurology.* 2015;78(4):594-605.

22. Yao S, Song J, Gao L, et al. Thalamocortical Sensorimotor Circuit Damage Associated with Disorders of Consciousness for Diffuse Axonal Injury Patients. *Journal of the neurological sciences.* 2015;356(1-2):168-174.

23. Vanhaudenhuyse A, Demertzi A, Schabus M, et al. Two distinct neuronal networks mediate the awareness of environment and of self. *Journal of cognitive neuroscience.* 2011;23(3):570-578.

24. Buckner RL, Andrews-Hanna JR, Schacter DL. The brain's default network: anatomy, function, and relevance to disease. *Annals of the New York Academy of Sciences.* 2008;1124:1-38.

25. D'Argembeau A, Feyers D, Majerus S, et al. Self-reflection across time: cortical midline structures differentiate between present and past selves. *Social cognitive and affective neuroscience.* 2008;3(3):244-252.

26. Mason MF, Norton MI, Van Horn JD, Wegner DM, Grafton ST, Macrae CN. Wandering minds: the default network and stimulus-independent thought. *Science.* 2007;315(5810):393-395.

27. Raichle ME, MacLeod AM, Snyder AZ, Powers WJ, Gusnard DA, Shulman GL. A default mode of brain function. *Proceedings of the National Academy of Sciences of the United States of America.* 2001;98(2):676-682.

28. Cauda F, Micon BM, Sacco K, et al. Disrupted intrinsic functional connectivity in the vegetative state. *Journal of neurology, neurosurgery, and psychiatry.* 2009;80(4):429-431.

29. Di Perri C, Bastianello S, Bartsch AJ, et al. Limbic hyperconnectivity in the vegetative state. *Neurology.* 2013;81(16):1417-1424.

30. Norton L, Hutchison RM, Young GB, Lee DH, Sharpe MD, Mirsattari SM. Disruptions of functional connectivity in the default mode network of comatose patients. *Neurology.* 2012;78(3):175-181.

31. Boly M, Tshibanda L, Vanhaudenhuyse A, et al. Functional connectivity in the default network during resting state is preserved in a vegetative but not in a brain dead patient. *Human brain mapping.* 2009;30(8):2393-2400.

32. Wu X, Zou Q, Hu J, et al. Intrinsic Functional Connectivity Patterns Predict Consciousness Level and Recovery Outcome in Acquired Brain Injury. *The Journal of neuroscience : the official journal of the Society for Neuroscience.* 2015;35(37):12932-12946.

33. Qin S, Duan X, Supekar K, Chen H, Chen T, Menon V. Large-scale intrinsic functional network organization along the long axis of the human medial temporal lobe. *Brain structure & function.* 2015.

34. Silva S, de Pasquale F, Vuillaume C, et al. Disruption of posteromedial large-scale neural communication predicts recovery from coma. *Neurology.* 2015;85(23):2036-2044.

35. Sharp D, Beckmann C, Greenwood R, et al. Default mode network functional and structural connectivity after traumatic brain injury. *Brain : a journal of neurology.* 2011;134(8):2233-2247.

36. Threlkeld ZD, Bodien YG, Rosenthal ES, et al. Functional networks reemerge during recovery of consciousness after acute severe traumatic brain injury. *Cortex; a journal devoted to the study of the nervous system and behavior.* 2018;106:299-308.

37. Fernandez-Espejo D, Soddu A, Cruse D, et al. A role for the default mode network in the bases of disorders of consciousness. *Annals of neurology.* 2012;72(3):335-343.

38. Herbet G, Lafargue G, de Champfleur NM, et al. Disrupting posterior cingulate connectivity disconnects consciousness from the external environment. *Neuropsychologia.* 2014;56:239-244.
