# Supplementary material for: Neural Connectivity Changes Facilitated by Familiar Auditory Sensory Training in Disordered Consciousness: A TBI Pilot Study
Source: Front Neurol. 2020 Oct 8;11:1027. doi: 10.3389/fneur.2020.01027 (PMC7578344; doi:10.3389/fneur.2020.01027)
Supplement: Supplementary file 2 [file Data_Sheet_2.DOCX]

B

**Medical Records Screened for Eligibility: N=50**

Written Consents: n=21

Excluded: n=29

Did Not Meet Inclusion Criteria (20/ 29):

6/21 Per treatment team, recovery of full consciousness was likely to have occurred prior to obtaining consent

5/21 Primary diagnosis of anoxia

2/21 More than one year from injury

2/21 Ventilator dependent

2/21 Not MRI compatible

2/21 Active seizures while on anticonvulsants

1/21 Remained medically unstable for 1 year

Refused Consent (4/29):

2/4 Family chose hospice placement

1/4 Potential for placebo

1/4 Duration of study too long

Other (5/29):

1/5 Expired prior to consent

1/5 No family or friends located

3/5 Residence too far from study sites

**Randomized: n=16**

Recruited from Acute Rehabilitation: 11/16 Recruited from Community: 5/16

**Allocated to FAST: n=8**

**Allocated to Placebo: n=8**

Loss to Follow-up = 0

Loss to Follow-up = 0

**Primary Neurobehavioral Analyses**: n=8

**SUPPLEMENT B: Consort Diagram for RCT & Imaging Sub-group for Pilot Study**

Screened Auditory Pathway & State of Consciousness: n=21

Withdrawn after Screening: n=5

1/21 Failed Auditory Pathway Screen

2/21 Recovered full consciousness

2/21 LAR chose hospice

**Protocol Compliance:**

Blinding: 1of 8 withdrawn by investigators due to complete un-blinding of group assignment

Treatment: 5/7: Received all 168 Placebo doses; 2/7 received 166 dose

**Protocol Compliance:**

Blinding: 8/8

Treatment: 7/8: Received all 168 FAST;

1/8 received164 doses

**Primary Neurobehavioral Analyses**: n=7

**Imaging Sub-group**: n = 4

Reasons for Exclusion: Motion-2; Signal drop from Shunt-1; Head Size-1

**Imaging Sub-group**: n=4

Reasons for exclusion: Motion- 3
