# Supplementary material for: Neural Connectivity Changes Facilitated by Familiar Auditory Sensory Training in Disordered Consciousness: A TBI Pilot Study
Source: Front Neurol. 2020 Oct 8;11:1027. doi: 10.3389/fneur.2020.01027 (PMC7578344; doi:10.3389/fneur.2020.01027)
Supplement: Supplementary file 3 [file Data_Sheet_3.DOCX]

**Neurobehavioral Measures: Reducing Within-subject Variability and Increasing Precision**

The Disorders of Consciousness Scale (DOCS) (publicly available at: <https://arch.library.northwestern.edu/collections/r781wg05z?locale=en>) is a measure of neurobehavioral function that starts with an initial systematic observation followed by administration of 25 sensory stimuli. Best responses to each stimulus are rated on a scale of 0 to 2 and total raw scores range from 0 (worst) to 50 (best). The DOCS-25 [^1^](#_ENREF_1) yields a reliable and valid measure of global neurobehavioral functioning with acceptable precision for clinical decision making for individual patients.[^1^](#_ENREF_1) The DOCS-25 also yields the DOCS Auditory-Language sub-scale score, which includes 6 of the 25 items with a raw score range of 0 to 18. These 6 Auditory-Language items include: functional use of toothbrush, orientation to environment, orientation to self, response to social greeting, response to name called aloud, and response to 1-step command. [^1^](#_ENREF_1) To account for multiple DOCS raters, we transformed DOCS-25 total raw scores to interval-level measures using Facets partial credit Rasch model.[^2^](#_ENREF_2) We then rescaled these logit measures to a 0 to 100 scale with higher measures indicating more neurobehavioral functioning.


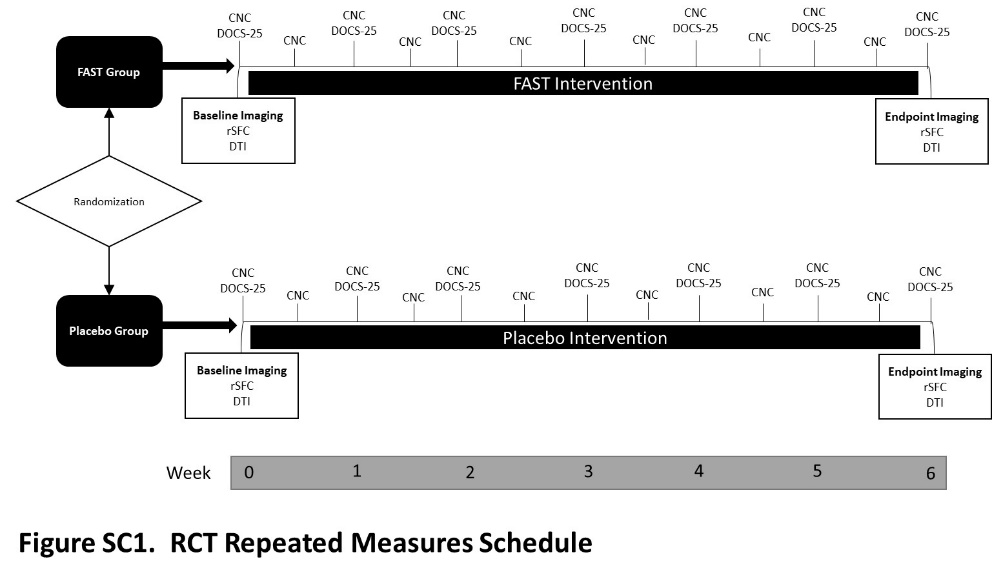
 To capture neurobehavioral change, the DOCS-25 and the CNC were repeated in the RCT, according to Figure SC1. The DOCS-25 is the only neurobehavioral scale for the DoC population that has established indices of responsiveness:[^3^](#_ENREF_3) (1) Cohen’s effect size (Improvers effect size: 0.45), (2) Standardized Response Mean: 1.3, and Clinically Important Differences (CID) have also been established: (3) Distribution-based CID: 2.6 – 6.6, and (4) Anchor-based CID: 8.6.[^3^](#_ENREF_3) Using methods similar to those published by Kozlowski et. al. (2016),[^4^](#_ENREF_4) we recently calculated conditional minimally detectable change indices (cMDC) for each pair of individual difference scores. For the range of DOCS change measures most patients in this RCT achieved, cMDCs were in the range of 2.0-2.5 units. Conditional MDCs reflect a gain or decline beyond measurement error, whereas CID indicates the smallest amount of clinically meaningful change.[^3^](#_ENREF_3)

The Coma Near Coma scale (CNC) also measures neurobehavioral function by responses to sensory stimuli, but by consistency of responses rather than by best response, as is used with the DOCS. Presence or absence of a specified behavior is scored as 0, 2 or 4. Lower scores indicate less consistency in manifesting a specified behavioral response. The total score ranges from 0 (consistently responsive) to 44 (extreme coma), with lower scores indicating more consistent behavioral responses indicative of arousal and awareness. [^5-7^](#_ENREF_5) [^8^](#_ENREF_8)^,^[^9^](#_ENREF_9)

**Nonlinear Warping Illustration: Reducing spatial variability between subjects while retaining each subject’s unique injury characteristics**

T1 images were normalized using the Voxel-based Morphometry (VBM) toolbox within Statistical Parametric Mapping (SPM8). Due to the heterogeneity of severe TBI, the nonlinear warping using DARTEL is used to spatially normalize the native T1 data to the MNI152 template space to reduce spatial variability between subjects. The BOLD and DTI data are linearly transformed to the native T1, and these transformations are concatenated to form a single warp to template space for these data.


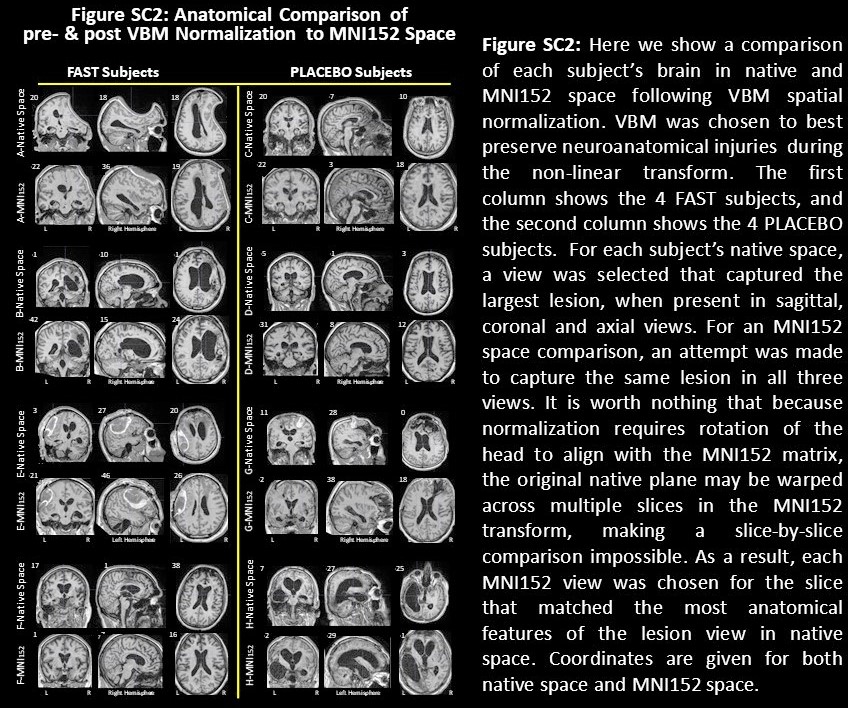
 We have found that the VBM toolbox (using DARTEL) is the most robust spatial normalization tool for the severe TBI population (and chronic stroke population) because it reduces between-subject spatial variability while preserving each subject’s neuroanatomical injuries. This is illustrated in the imaging sub-group participants in Figure SC2, which depicts how VBM reduces spatial variability while preserving anatomical injuries for each participant.

**Mixed-effects Linear Modeling Procedures for z, FA and MD: Modeling Raw Values**

To account for within- and between-subject variability and missing values, we used mixed -effects linear models (MLM) to predict/estimate imaging values (i.e., z, FA, MD). Imaging is an indirect measure of brain activity, but the term ‘estimated imaging values’ here refers to imaging values predicted from MLM modeling of the raw imaging values. Given the heterogeneity of severe TBI, this modeling approach was used because the predicted imaging values account for within- and between-subject variability. Using this model, a random effect for each subject was generated, and all predicted imaging values were used as estimates of imaging values.

We used the estimated imaging values in all analyses because each value accounts for within-and between-subject variability. Since MLM can also handle missing at random data, and it can incorporate variation within a multi-level hierarchy, we also used MLM predicted values to impute values for values classified as missing. Regions of interests (ROI) were classified as missing when we had low confidence (e.g., lack of reliable landmarks to draw ROI); we chose to define any non-distinguishable or absent/not-present ROI as missing data. While some ROIs missing at baseline were distinguishable at endpoint, given recovery, the majority that were missing at baseline continued to be missing at endpoint. Since no subject had a lobectomy, it is likely that these non-distinguishable ROIs are not actually absent. Thus, when a subject had an absent/not present or indiscernible substrate, MLM derived estimates of change in FA for those substrates were imputed. This means that for tracts with more than one missing data point, the results are based largely on estimates derived according to other subjects’ actual values and the other subjects’ random effects. That is, our approach was to estimate (i.e., predict) the missing value for a given subject based on the average of other subjects nested in the same group (FAST or Placebo) utilizing MLM-based predicted values.

**Validation of MLM Estimated Values Imputed for Missing Brain Connectivity Metrics**

We validated the MLM procedures conducted to estimate missing values. This was done by verifying findings that were based on the predicted/estimated imaging values. Specifically, we verified the positive correlation between increasing FA for the Left ILF and improving DOCS Auditory-Language skills. We chose to verify this finding because the Placebo group includes a subject defined by the authors as missing the Left ILF at baseline and endpoint (Subject H; Also see Figure 4 in main manuscript and Figure SC1 above). Their left ILF was not discernible or was not present/absent; therefore, this tract was classified by authors as missing data for Subject H.

One goal of the verification was to understand the error within the estimated/predicted FA values for the left ILF for Subject H. To understand the error within the estimates, we reran all mixed-effects linear models (MLM) for all subjects but deleted subject H from the analysis. That is, we computed the model-based expected values (i.e., predicted values/estimates) to compare the predicted/estimated value with the actual or observed value. We did this for each of the three subjects separately and produced an overall measure where we combined all three subjects in the Placebo group (i.e., where subject H was ignored). We computed two measures to examine discrepancy between the actual and predicted/estimated values:

(i) Mean Absolute Error (MAE) = Mean of [Abs(observed-predicted)]

(ii) Mean Absolute Relative Error (MARE) = Mean [Abs(observed-predicted)/Observed]

| **Table SC1. Average Error for Predicted Values/Imputed Estimates** | | |
| --- | --- | --- |
|  | **MARE** | **MAE** |
| Subject G | 0.242506 | 0.108477 |
| Subject F | 0.059735 | 0.023531 |
| Subject E | 0.459664 | 0.132003 |
| **Overall** | **0.253969** | **0.088004** |

Table SC1 below provides the MARE and MAE values for each subject based on the baseline and endpoint measures. The last row of the table provides the overall/average MARE and MAE values (based on all three subjects). Each subject has two predicted/estimated values, one at baseline and another at endpoint. First, we computed the average errors of each subject that are provided in the MAE column. Next, we computed the average of these averages, which is indicated in the overall row within the MAE column. The MAE overall average absolute error (0.088004), based on two time points for all three subjects, indicates small average error for the predicted/estimated values. We computed the MARE for each subject and overall in a similar manner. The overall MARE finding (0.253969) also indicates small average error for the predicted/estimated imaging values.

While the baseline and endpoint values for actual and predicted/estimated are dependent, we wanted to further understand the magnitude of the difference between the actual and predicted (estimated) values for additional insights about accuracy of the predicted values (i.e., estimates imputed for missing values). Therefore, we also examined the agreement between actual overall values and predicted values, overall, using Chi Square. We computed the chi^2 value based on the total of [(actual-predicted)^2/ predicted] and test it with 5 degrees of freedom. The results indicate no significant difference (chi-square statistic = .1556, df = 5, p = 0.999).

**Computing Test Statistics, Ordering Test Statistics & Computing Ordered p-values:**

Notations:

- P = Baseline (B) and Endpoint (EP) are Permuted
- NP = B and EP are not permuted.
- Thus, for subject J, if B and EP are permuted, we denote this as JP. When B and EP for subject J are not permuted, we denote this as JNP.

For a total of 3 subjects there are a total of 8 permutations, as follows:

- (1NP, 2NP, 3NP) + (1P, 2NP, 3NP) + (1NP, 2P, 3NP) + (1P, 2NP, 3P) + (1P, 2NP, 3P) + (1NP, 2P, 3P) + (1P, 2P, 3NP) + (1P, 2P, 3P) = 8 permutations.

Computation of Original tests statistic, t1, and other Permuted test statistics, t2-t8, are done for each of the above 8 permutations:

- These test statistics (Original and Permuted) are then produced: t1, t2, t3, t4, t5, t6, t7, t8
- Ordering the test statistics is done next to address the question of where the position of the original t1 test statistic is located with respect to the permuted test statistics. Specifically, the permuted test statistics are ordered from the smallest to the largest and denoted with parentheses as follows:
  - t(1), t(2), t(3), t(4), t(5), t(6), t(7), t(8), hence t(1)$\leq$t(2) $\leq$t(3) $\leq$t(4)$\leq$t(5)$\leq$t(6)$\leq$t(7)$\leq$ t(8).
    - Thus, t(8) is the largest permuted test statistic and t(1) is the smallest permuted test statistic.
- We assumed no distributions on these test statistics, so the cut-off values were not known; hence, exact p-values could not be computed (as it requires a known distribution to compute the p-value).
  - See bullet below for how ordered p-values were computed to address the issue of unknown cut-off values.
- The original test statistic is t1 (i.e., without any permutation, thus it is denoted without parentheses). If t1 is the largest, (i.e. t1= t(8)), then it will have the smallest p-value for a right-sided test (even though we do not know exact p-value), hence providing us strong confidence that, in reality, no other possible p-value is smaller than what we have observed. Since the largest test statistic will yield the smallest p-value, we use this to determine significance. Examples are as follows:
  - For smaller sample sizes (n), the exact ordered p-values for the permutation tests, such as those reported in Table 2 of the main manuscript, were computed using the number of ordered statistics > t1 (the original test statistic) and the total number of permuted test statistics.
    - For example, when n = 4 subjects and there are 3 ordered test statistics > t1, then the test will have a p-value = 3/2^4^ = 3/16 = 0.19.
  - This approach to computing ordered p-values:
    - Does not compute p-values based on any distributional assumption, and
    - Does not compare those with the adjusted alpha value (like Bonferroni).

**Controlling for False Discovery using Permutated t-tests of significance**

To test significance of change (Endpoint – Baseline) within and between the FAST and Placebo groups, we used permutation because it is based on minimal assumptions regarding the distribution of the test statistic. [^10^](#_ENREF_10) Permutation tests were used because the small sample size indicates the importance of minimizing assumptions regarding the distribution of the test statistic.[^10^](#_ENREF_10) That is, under the null hypothesis of no difference, permuted t-test statistics were obtained by calculating all possible values under rearrangements of the labels on the observed data points. The cut-off value(s) is determined by considering a certain percentile point of all permuted ordered statistics. Thus, this procedure does not need to compute the p-value of the test statistic which always depends on a distributional assumption of the null test statistic. Testing for significance using this approach maintains validity of statistical inferences when examining a small non-random sample of RCT participants who were selected according to quality of imaging data.

To determine significant between-group differences, two study groups were permuted. For within -group (e.g. baseline *vs* endpoint) comparisons, we permuted the pair of observations nested within the same subject (e.g. interchanging the roles of baseline and endpoint). Thus, for a total of n subjects nested within a group with no missing values, we had 2^n^ permuted paired t statistics.

For multiple comparisons, we used the false discovery rate procedure (See Figure SC3 for illustration) because it is not overly conservative, and we used a threshold of .20 because in the early stage of research we did not want to obscure changes that could provide insights for future research. This means, though, that out of every 100 discoveries, we would not expect to make more than an average of 20 false discoveries.[^11^](#_ENREF_11) In other words, the expectation for every 5 discoveries would be only one false discovery and 4 true discoveries. At this stage in the research, it is important to detect meaningful differences, and a q smaller than .20 could possibly miss meaningful effects. Future research with larger sample sizes can restrict q without threat of missing a meaningful difference.

**Figure SC3. Example of FDR based on Permuted p-values for multiple comparisons**

Using the DTI data, the FDR approach ordered all 19 white matter tract p-values, p_(1)_ ≤ p_(2)_ ≤ ….≤ p_(19)_ , and compared the i^th^ ordered p_(i)_ with $\frac{i q}{19},$ starting from p_(19)_ and then repeated the process with p_(18),_ p_(17) …._ p_(r)_ until we had p_(r)_ ≤ $\frac{r q}{19}$.

For such an r, all hypotheses related to p_(1)_, p_(2),_……., p_(r)_ were rejected. This is an adaptive adjustment procedure, where q (instead of α=.05) was divided by the total number of hypotheses, but at each step, $\frac{q}{19}$ was multiplied by the corresponding step number *i*.

The significance of the findings based on raw data (all results from significance testing based on raw data values are provided in Tables C1 and C2 in Supplement C) may vary from those obtained from estimated values (all results from significance testing based on estimated values are presented in Tables E1 and E2 in Supplement E), but only one finding (FAST group FA Right ILF) differs in terms of direction of change. For the FAST group, the FA based on raw data significantly decreases from .383 to .377 (p < 0.01), and with estimated values it increases from .38 to .41 such that it is significantly (p < 0.01) different at endpoint. To be prudent, we do not consider the Right ILF finding for the FAST group when interpreting the results.

When comparing significance testing results based on raw values versus estimated values, there were three findings that differ in terms of significance at endpoint. Findings that are significantly different when using the estimated values, but that are not significant based on raw data values, are the FA of the Right AF (FAST) and FA of the Right SFOF (Placebo). Findings not significantly different when using the estimated values, and that are significantly different when using raw data values, involve for the FA of Right ILF (Placebo). To be transparent, we summarize these ‘discrepancies’ here, but these discrepancies are not surprising as the t-tests we used for raw data are localized, whereas estimated based results depend on the surrounding locations, and hence may be different from the raw data results. Also, results based on raw data do not remove noise, whereas MLM results borrow strength from multiple sources, smooths various types of heterogeneities, and provide efficient estimates of model parameters.

**Standardizing and Scaling FA for Left ILF: Verification of Positive Correlation Finding**

A meaningful clinical treatment effect is indicated by a positive correlation between changes in structural connectivity and neurobehavioral gains. Yet, the imaging sub-group includes subjects with varying lengths of white fiber tracts. Therefore, we decided to conduct a verification analysis to determine if the behavioral correlation results would change if we standardized the amount of substrate in the same space while controlling for individual brain size based on length. For this verification analysis, we examined the validity of the main positive correlation finding that the Left ILF FA change is positively correlated with DOCS Auditory-Lang change. As illustrated in Figure 4 of the main manuscript, we verified this finding by computing the FA value in normalized ROIs that were limited to the smallest segment across subjects to restrict the analysis to the same white matter. We adjusted this ROI to standardize the amount of substrate in the same space controlling for individual brain size based on length. We then used these adjusted Left ILF ROIs to extract FA values. We used the adjusted FA metrics in Mixed Linear Effects models (MLM) to compute FA change from baseline to endpoint. We used these MLM derived and adjusted FA change measures to re-examine correlation with DOCS Auditory-Language measures. The procedural steps that were completed are:

1. Computation of Adjusted Left ILF ROIs was done as follows:
   1. 14 voxel least common denominator: The length of the Left ILF was limited for all subjects according to the shortest ILF (Subject G = 14 voxels) in the sample
   2. Drawing 14 voxel ROI/Left ILF: The same morphological landmark for each subject was used as the posterior starting point for drawing (i.e., posterior pole)
   3. Standardizing the FA Values for the 14 voxel Left ILF for each subject according to the normal persons' brain size (Anterior(A) to Posterior (P) intracranial length): this was done for each subject by adjusting/scaling each subject’s 14 voxel Left ILF by the Anterior to Posterior normal (MNI) whole brain length (intracranial) using this formula:

[(Subject’s A-P) / (Normal A-P) * 14 voxels] = Scaled L ILF for each subject

**(A – P) =** Intracranial whole brain Anterior (A) - Posterior (P) difference

**Normal A-P =** Average MNI Intracranial whole brain A- P

- 1. Meaning of extracted FA values based on scaled L ILF: The extracted FA values reflect each subject having the same amount of substrate in the ROI examined (Left ILF) within the same space controlling for individual brain size based on length of health persons' brain

1. To verify the presence of a positive correlation, we:
   1. First inspected/compared the FA for the Left ILF/ROI derived from native space (where each subject has a unique Left ILF length) with Adjusted Left ILF FAs
   2. To verify finding, we then computed Adjusted FA change in Left ILF (from Baseline to Endpoint) by re-running the same mixed -effects linear models (MLM). To verify correlation finding, we then used the MLM derived and adjusted FA change to -compute the correlation between Adjusted FA change and DOCS Auditory-Language Change.

**Citations for Supplement C:**

1. Pape TL, Mallinson T, Guernon A. Psychometric properties of the disorders of consciousness scale. *Archives of physical medicine and rehabilitation.* 2014;95(9):1672-1684.

2. Linacre M. *Many Facets Rasch Measurement.* Chicago, IL: Mesa Press, University of Chicago; 1994.

3. Mallinson T, Pape TL, Guernon A. Responsiveness, Minimal Detectable Change, and Minimally Clinically Important Differences for the Disorders of Consciousness Scale. *The Journal of head trauma rehabilitation.* 2016;31(4):E43-51.

4. Kozlowski AJ, Cella D, Nitsch KP, Heinemann AW. Evaluating Individual Change With the Quality of Life in Neurological Disorders (Neuro-QoL) Short Forms. *Archives of physical medicine and rehabilitation.* 2016;97(4):650-654 e658.

5. Rappaport M, Hall K, Hopkins HK, Belleza T. Evoked potentials and head injury. 1. Rating of evoked potential abnormality. *Clinical EEG.* 1981;12(4):154-166.

6. Rappaport M. Brain evoked potentials in coma and the vegetative state. *Journal of Head Trauma Rehabilitation.* 1986;1:15-29.

7. Rappaport M. Electrophysiological Assessment. In: HornL J, Zasler ND, eds. *Medical rehabilitation of traumatic brain injury.* Philadelphia, PA: : Hanley & Belfus, Inc.; 1996:271-316).

8. Rappaport M, Dougherty, A., & Kelting, D. Evaluation of Coma & Vegetative States. *Archives of Physical Medicine & Rehabilitation.* 1992;73:628-634.

9. Rappaport M. The Disability Rating Scale/Coma-Near-Coma Scale in evaluating severe head injury. *Neuropsychological Rehabilitation.* 2005;15(3/4):442-453.

10. Nichols TE, Holmes AP. Nonparametric permutation tests for functional neuroimaging: a primer with examples. *Human brain mapping.* 2002;15(1):1-25.

11. Bhaumik DK, Roy A, Lazar NA, et al. Hypothesis testing, power and sample size determination for between group comparisons in fMRI experiments. *Statistical methodology.* 2009;6(2):133-146.
