# Supplementary material for: Neural Connectivity Changes Facilitated by Familiar Auditory Sensory Training in Disordered Consciousness: A TBI Pilot Study
Source: Front Neurol. 2020 Oct 8;11:1027. doi: 10.3389/fneur.2020.01027 (PMC7578344; doi:10.3389/fneur.2020.01027)
Supplement: Supplementary file 4 [file Data_Sheet_4.DOCX]

**Table SD1 Raw Z values FAST Group by Networks**

**Table SD2 Raw Z values Placebo Group by Time points and Networks**

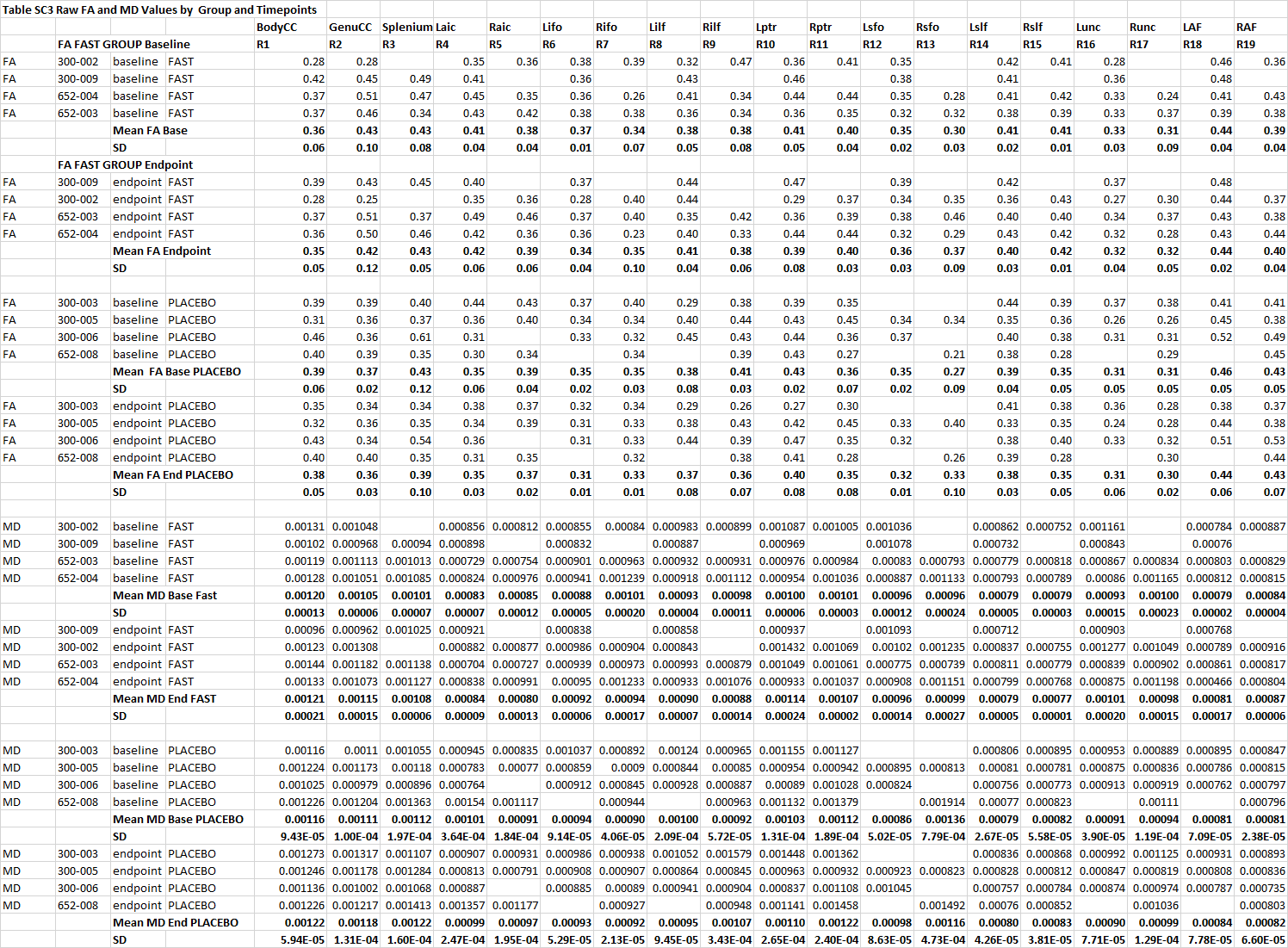


**Table SD3 Raw FA and MD Values by Groups and Timepoints**

| Table SD4. Significance Testing with Raw Mean Z Values: Within & Between Networks | | | | | | | | | | | | |
| --- | --- | --- | --- | --- | --- | --- | --- | --- | --- | --- | --- | --- |
|  | **FAST Group** | | | **Placebo Group** | | | | | **FAST *vs* Placebo** | | |  |
|  | **A** | **B** | **C** | **D** | **E** | | **F** | **G** | | **H** |  |  |
|  | **Mean Correlations (Z Values)**  **(n= sample sizes)** | | **(End – Base)** | **Mean Correlations (Z Values)**  **(n= sample sizes)** | | | **(End – Base)** | **Baseline** | | **Endpoint** |  |  |
|  | **Baseline** | **Endpoint** | **Ordered p** ^i^ | **Baseline** | | **Endpoint** | **Ordered p ^t^** | **Ordered p ^i^** | | **Ordered p** ^i^ |  |  |
| AN | 0.30 (n = 3) | 0.29 (n = 4) | 0.13 | 0.27 (n = 4) | | 0.27 (n = 4) | 0.44 | 0.31 | | 0.36 |  |  |
| DMN | 0.34 (n = 3) | 0.28 (n = 4) | **<0.00** | 0.29 (n = 4) | | 0.29 (n = 4) | 0.44 | 0.28 | | 0.41 |  |  |
| LN | 0.51 (n = 3) | 0.41 (n = 4) | **<0.00** | 0.44 (n = 4) | | 0.42 (n = 4) | 0.44 | 0.31 | | 0.50 |  |  |
| SN | 0.44 (n = 3) | 0.30 (n = 4) | **<0.00** | 0.42 (n = 4) | | 0.41(n = 4) | 0.44 | 0.46 | | **0.03 (F < P)** |  |  |
| AN-DMN | 0.60 (n = 3) | 0.67 (n = 4) | 0.25 | 0.74 (n = 4) | | 0.67 (n = 4) | 0.13 | 0.83 | | 0.50 |  |  |
| AN-LN | 0.53 (n = 3) | 0.29 (n = 4) | 0.13 | 0.44 (n = 4) | | 0.53 (n = 4) | **<0.00** | 0.23 | | **0.03 (F < P)** |  |  |
| AN-SN | 0.26 (n = 3) | 0.31 (n = 4) | **<0.00** | 0.32 (n = 4) | | 0.30 (n = 4) | 0.44 | 0.26 | | 0.43 |  |  |
| DMN-LN | 0.75 (n = 3) | 0.65 (n = 4) | 0.25 | 0.54 (n = 4) | | 0.56 (n = 4) | 0.31 | **0.05 (F > P)** | | 0.21 |  |  |
| DMN-SN | 0.55 (n = 3) | 0.55 (n = 4) | 0.13 | 0.47 (n = 4) | | 0.43 (n = 4) | 0.44 | 0.34 | | 0.24 |  |  |
| LN-SN | 0.58 (n = 3) | 0.76 (n = 4) | 0.13 | 0.74 (n = 4) | | 0.68 (n = 4) | 0.31 | 0.66 | | 0.24 |  |  |
| Black shaded cells = significant ordered p value ^i^ where significance testing using permutation accounted for multiple comparisons using FDR q = .20 b; F > P and F < P = FAST group mean is significantly greater or less than Placebo mean; AN = Attention Network; B = Baseline; DMN = Default Mode Network; E = Endpoint; FAST = Familiar Auditory Sensory Training; i = independent sample permuted t-tests conducted with mean z values derived from Fisher z transform of raw data. LN = Language Network; Placebo = Silence; SN = Salience Network ; Ordered p = independent sample permuted t-tests conducted with mean estimated z values; Black = ordered p ≤ 0.05 | | | | | | | | | | | | |

| **Table SD5. Mean FA and MD Values at Baseline and Endpoint, Significance of Change (Endpoint-Baseline) Within & Between Groups** | | | | | | | | | | | | | | |
| --- | --- | --- | --- | --- | --- | --- | --- | --- | --- | --- | --- | --- | --- | --- |
|  | **FAST FA** | | | **Placebo FA** | | | **FAST vs Placebo**  **End-Base p** | **FAST MD** | | | **Placebo FA** | | | **FAST vs Placebo**  **End (E)-Base (B) p** |
|  | **Mean FA (SD)** | | **End (E)-Base (B) p (n)** | **Mean FA (SD)** | | **End (E)-Base (B) p (n)** |  | **Mean MD (SD)** | | **End (E)-Base (B) p (n)** | **Mean MD (SD)** | | **End (E)-Base (B) p (n)** |  |
|  | **Base** | **End** |  | **Base** | **End** |  |  | **Base** | **End** |  | **Base** | **End** |  |  |
| **Body of CC** | 0.357 (0.059) | 0.351 (0.051) | 0.20 (n = 4_)_ | 0.388 (0.06) | 0.375 (0.05) | 0.125  (n = 4) | 0.414 | 0.0012 (0.00013) | 0.00124 (0.00021) | **0.40**  **(n = 4)** | 0.00116 (0.00009) | 0.00122 (0.00006) | 0.06  (n = 4) | 0.41 |
| **Genu of CC** | 0.426 (0.103) | 0.422 (0.12) | 0.53  (n = 4) | 0.374 (0.02) | 0.361 (0.03) | 0.062  (n = 4) | 0.45 | 0.00105 (0.00006) | 0.00113 (0.00015) | **0.00**  **(n = 4)** | 0.00111 (0.0001) | 0.00118 (0.00013) | 0.06  (n = 4) | 0.31 |
| **Splenium of CC** | 0.432 (0.079) | 0.425 (0.052) | **<0.01**  **(n = 3 _B_,3 _E_)** | 0.432 (0.117) | 0.394 (0.096) | **<0.01**  **(n = 4)** | 0.38 | 0.00101 (0.00007) | 0.0011 (0.00006) | **<0.01**  **(n = 3 _B_,3 _E_)** | 0.00112 (0.0002) | 0.00122 (0.00016) | **<0.01**  **(n = 4)** | **0.37** |
| **Left Ant IC Limb** | 0.41 (0.044) | 0.417 (0.057) | 0.4  (n = 4) | 0.352 (0.063) | 0.348 (0.028) | 0.437  (n = 4) | 0.37 | 0.00083 (0.00007) | 0.00084 (0.00009) | **0.13**  **(n = 4)** | 0.00101 (0.00036) | 0.00099 (0.00025) | **<0.01**  **(n = 4)** | 0.26 |
| **Right Ant IC Limb** | 0.379 (0.039) | 0.391 (0.056) | 0.20  (n = 3 _B_,3 _E_) | 0.39 (0.044) | 0.369 (0.02) | 0.250  (n = 3 _B_,3 _E_) | 0.48 | 0.00085 (0.00012) | 0.00087 (0.00013) | 0.13  (n = 3 _B_,3 _E_) | 0.00091 (0.00018) | 0.00097 (0.0002) | 0.38  (n = 3 _B_,3 _E_) | 0.37 |
| **Left**  **IFOF** | 0.37 (0.012) | 0.343 (0.043) | **<0.01**  **(n = 4)** | 0.346 (0.017) | 0.312 (0.008) | **<0.01**  **(n = 3 _B_,3 _E_)** | 0.34 | 0.00088 (0.00005) | 0.00093 (0.00006) | **<0.01**  **(n = 4)** | 0.00094 (0.00009) | 0.00093 (0.00005) | 0.13  (n = 3 _B_,3 _E_) | 0.17 |
| **Right**  **IFOF** | 0.344 (0.069) | 0.346 (0.1) | 0.40  (n = 3 _B_,3 _E_) | 0.35 (0.032) | 0.328 (0.01) | 0.120  (4) | 0.43 | 0.00101 (0.0002) | 0.00104 (0.00017) | **<0.01**  **(n = 3 _B_,3 _E_)** | 0.0009 (0.00004) | 0.00092 (0.00002) | 0.38  (n = 4) | 0.43 |
| **Left**  **ILF** | 0.382 (0.05) | 0.407 (0.043) | 0.13  (n = 4 ) | 0.382 (0.085) | 0.368 (0.078) | **<0.01**  **(n = 3 _B_,3 _E_)** | **0.17** | 0.00093 (0.00004) | 0.00091 (0.00007) | **0.31**  **(n = 4)** | 0.001 (0.00021) | 0.00095 (0.00009) | 0.13  (n = 3 _B_,3 _E_) | 0.40 |
| **Right**  **ILF** | 0.383 (0.075) | 0.377 (0.064) | <0.01  (n = 3 _B_,2 _E_) | 0.409 (0.032) | 0.362 (0.074) | **<0.01**  **(n = 4)** | **0.11** | 0.00098 (0.00011) | 0.00098 (0.00014) | **<0.01**  **(n = 3 _B_,3 _E_)** | 0.00092 (0.00006) | 0.00107 (0.00034) | 0.31  (n = 4) | 0.06 |
| **Left**  **PTR** | 0.405 (0.051) | 0.389 (0.08) | **<0.01**  **(n = 4)** | 0.425 (0.022) | 0.395 (0.084) | 0.125  (n = 4) | 0.4 | 0.001 (0.00006) | 0.00109 (0.00024) | 0.187  (n = 4) | 0.00103 (0.00013) | 0.0011 (0.00027) | 0.13  (n = 4) | 0.29 |
| **Right**  **PTR** | 0.399 (0.044) | 0.398 (0.033) | 0.25  (n = 3 _B_,3 _E_) | 0.358 (0.072) | 0.346 (0.077) | 0.250  (n = 4) | 0.36 | 0.00101 (0.00003) | 0.00106 (0.00002) | **<0.01**  **(n = 3 _B_,3 _E_)** | 0.00112 (0.00019) | 0.00122 (0.00024) | 0.250  (n = 4) | 0.11 |
| **Left**  **SFOF** | 0.351 (0.022) | 0.358 (0.034) | **<0.01**  **(n = 4)** | 0.353 (0.024) | 0.322 (0.009) | 0.250  (n = 2 _B_,2 _E_) | 0.08 | 0.00096 (0.00012) | 0.00095 (0.00014) | **<0.01**  **(n = 4)** | 0.00086 (0.00005) | 0.00098 (0.00009) | 0.25  (n = 2 _B_,2 _E_) | 0.20 |
| **Right SFOF** | 0.302 (0.031) | 0.365 (0.087) | **<0.01**  **(n = 2 _B_,3 _E_)** | 0.274 (0.094) | 0.329 (0.101) | 0.250  (n = 2 _B_,2 _E_) | 0.23 | 0.00096 (0.00024) | 0.00104 (0.00027) | 0.25  (n = 2 _B_,3 _E_) | 0.00136 (0.00078) | 0.00116 (0.00047) | 0.25  n = 2 _B_,2 _E_) | 0.17 |
| **Left**  **SLF** | 0.406 (0.02) | 0.402 (0.03) | 0.375  (n = 4) | 0.393 (0.039) | 0.379 (0.032) | 0.312  (n = 4) | 0.33 | 0.00079 (0.00005) | 0.00079 (0.00005) | 0.43  (n = 4) | 0.00079 (0.00003) | 0.0008 (0.00004) | 0.19  (n = 4) | 0.09 |
| **Right**  **SLF** | 0.406 (0.013) | 0.416 (0.014) | 0.125  (n = 3 _B_,3 _E_) | 0.353 (0.049) | 0.352 (0.054) | 0.125  (n = 4) | 0.17 | 0.00079 (0.00003) | 0.00077 (0.00001) | 0.13  (n = 3 _B_,3 _E_) | 0.00082 (0.00006) | 0.00083 (0.00004) | 0.13  (n = 4) | 0.11 |
| **Left**  **UF** | 0.325 (0.034) | 0.325 (0.044) | 0.375  (n = 4) | 0.314 (0.053) | 0.308 (0.061) | 0.125  (n = 3 _B_,3 _E_) | 0.4 | 0.00093 (0.00015) | 0.00097 (0.0002) | 0.06  (n = 4) | 0.00091 (0.00004) | 0.0009 (0.00008) | 0.13  n = 3 _B_,3 _E_) | 0.14 |
| **Right**  **UF** | 0.307 (0.088) | 0.318 (0.046) | **<0.01**  **(n = 2 _B_,3 _E_)** | 0.31 (0.051) | 0.298 (0.02) | 0.250  (n = 4) | **0.47** | 0.001 (0.00023) | 0.00105 (0.00015) | **<0.01**  **(n = 2 _B_,3 _E_)** | 0.00094 (0.00012) | 0.00099 (0.00013) | 0.06  (n = 4) | 0.23 |
| **Left**  **AF** | 0.436 (0.042) | 0.444 (0.024) | 0.25  (n = 4) | 0.458 (0.054) | 0.442 (0.062) | **<0.01**  **(n = 3 _B_,3 _E_)** | 0.13 | 0.00079 (0.00002) | 0.00072 (0.00017) | 0.43  (n = 4) | 0.00081 (0.00007) | 0.00084 (0.00008) | **<0.01**  **(n = 3 _B_,3 _E_)** | 0.26 |
| **Right**  **AF** | 0.390 (0.037) | 0.399 (0.035) | 0.125  (n = 3 _B_,3 _E_) | 0.432 (0.049) | 0.430  (0.073) | 0.312  (n = 4) | 0.40 | 0.00084 (0.00004) | 0.00085 (0.00006) | 0.25  (n = 3 _B_,3 _E_) | 0.00081 (0.00002) | 0.00082 (0.00007) | 0.38  (n = 4) | .46 |
| **Black** = significant; **AF** = Arcuate Fasciculus; **Ant** = Anterior; **CC** = corpus callosum; **IC** = internal capsule; **IFOF & SFOF** = Inferior & Superior Fronto-Occipital Fasciculus; **ILF** = Inferior Longitudinal Fasciculus; **PTR** = Posterior Thalamic Radiation; S**D** = Std Deviation; **SLF** = Superior Longitudinal Fasciculus; **UF** = Uncinated Fasciculus; **Sample Sizes**: n = 4 at each time-point unless specified as **n_B_** =baseline and **n_E_ =** Endpoint | | | | | | | | | | | | | | |
