# Supplementary material for: Neural Connectivity Changes Facilitated by Familiar Auditory Sensory Training in Disordered Consciousness: A TBI Pilot Study
Source: Front Neurol. 2020 Oct 8;11:1027. doi: 10.3389/fneur.2020.01027 (PMC7578344; doi:10.3389/fneur.2020.01027)
Supplement: Supplementary file 5 [file Data_Sheet_5.DOCX]

| **Table SE1. Baseline Differences of RCT Participants Excluded *vs* Included in Pilot Study Imaging Sub-group** | | | | | | |
| --- | --- | --- | --- | --- | --- | --- |
|  | **RCT Sample by Inclusion vs Exclusion from Pilot Study Imaging Sub-Group** | | | **Within Pilot Study Imaging Sub-group** | | |
|  | **A** | **B** | ***C*** | **D** | **E** | ***F*** |
|  | **Excluded (n = 7)** | **Included (n= 8)** | ***p*** | **FAST (n = 4)** | **Placebo (n = 4)** | ***p*** |
| Age at Injury | 29.7; SD: 6.5 | 39.8; SD: 12.4 | **.07** | 40.5; SD: 13.7 | 38.8; SD: 12.7 | .88 |
| Male Gender | 5 (71%) | 7 (88%) | .44 | 3 (75%) | 4 (100%) | .29 |
| Race/Ethnic Group:  -White  -Black  -Hispanic  -Asian | 3 (42%)  2 (29%)  2 (29%)  0 (0%) | 4 (50%)  1 (12%)  1 (13%)  2 (25%) | .78* | 2 (50%)  1 (25%)  0 (0%)  1 (25%) | 2 (50%)  0 (0%)  1 (12%)  1 (13%) | 1.0* |
| Education Achieved at Injury:  -High School (HS) Graduate or GED  -Some College, No Degree  -Bachelor Degree  -Graduate or Professional Degree | 3 (43%)  3 (43%)  1 (14%)  0 (0%) | 2 (25%)  1 (12%)  3 (38%)  2 (25%) | .46 ** | 2 (50%)  0 (0%)  1 (25%)  1 (25%) | 0 (0%)  1 (25%)  2 (50%)  1 (25%) | .10** |
| Etiology/Cause of Injuries:  -Automobile  -Motorcycle and Snowmobile  -Assaults and falls  -Pedestrian struck by vehicle | 5 (71%)  2 (29%)  0 (0%)  0 (0%) | 1 (12%)  2 (25%)  3 (38%)  2 (25%) | **.01^+^** | 0 (0%)  1 (25%)  2 (50%)  1 (25%) | 1 (25%)  1 (25%)  1 (25%)  1 (25%) | 0.47^+^ |
| Days between Injury and Study Enrollment | 60.7; SD:49.5 | 77.9; SD: 37.6 | .46 | 77.8; SD: 38.2 | 78.0; SD: 42.9 | .99 |
| Days between Injury and Study Baseline | 70.7; SD: 59.9 | 104.0; SD:45.4 | .25 | 117.0; SD: 56.2 | 91.0; SD: 34.5 | .46 |
| Injury Severity Score at Injury | 34.5; SD:13.2 | 28.9; SD:13.4 | .45 | 31.3; SD: 14.1 | 26.5; SD: 14.3 | .65 |
| Highest AIS Score at Injury | 4.5; SD:0.84 | 4.9; SD:0.35 | .27 | 4.8; SD: 0.5 | 5.0; SD: 0.0 | .39 |
| CNC Baseline Measure | 54.73; SD:2.0 | 52.5; SD:3.4 | .15 | 26.0; SD: 5.4 | 21.5; SD: 4.1 | .23 |
| DOCS-25 Baseline Measure | 53.2; SD: 10.2 | 57.9; SD: 8.9 | .36 | 57.1; SD: 11.9 | 60.0; SD: 5.1 | .67 |
| Baseline Clinical States  - Vegetative State (VS)  - Minimally Conscious State (MCS) | 2 (29%)  5 (71%) | 3 (38%)  5 (62%) | .71 ^++^ | 2 (50%)  2 (50%) | 1 (25%)  3 (75%) | .47 ^++^ |
| Total # of Medical Comorbidities | 1.6; SD: .54 | 1.5: SD: .54 | .80 | 1.8; SD: .50 | 1.3; SD: .50 | .21 |
| # of Lesions - All Lobes | 9.0; SD:3.2 | 8.5; SD: 2.7 | .77 | 6.8; SD: 2.2 | 10.3; SD: 2.1 | **.06** |
| # of Areas with Contusions | 7.6; SD:4.1 | 6.0; SD: 3.6 | .47 | 3.7; SD: 2.6 | 8.3; SD: 2.9 | **.06** |
| # of Areas with Subdural Hematomas | 2.4; SD: 3.4 | 1.4; SD: 2.2 | .52 | 2.8; SD: 2.5 | 0.0; SD: 0.0 | .11 |
| # of Mass Lesions | 2.4; SD:3.4 | 1.3; SD: 2.1 | .46 | 1.3; SD: 1.9 | 1.3; SD: 2.5 | .99 |
| # of Areas with SAH | 2.4; SD:3.6 | 2.3; SD: 3.4 | .94 | 2.3; SD: 2.6 | 2.3; SD: 4.5 | .99 |
| # of Areas with Diffuse Axonal Injury | .8.6; SD: 3.2 | 5.9; SD: 3.6 | .19 | 4.3; SD: 2.6 | 7.5; SD: 4.1 | .23 |
| Active Seizures; Receiving Prophylaxis | 2 (29%) | 0 (0%) | .10 | 0 (0%) | 0 (0%) | NS |
| **Usual Care Rehabilitation Treatments and Services Received During RCT** | | | | | | |
| CNS Modulator* | 3/4 (75%)^ | 6/7 (86%)^ | .66 | 3 (75%) | 3 (100%) | .57 |
| Benzodiazepine* | 3/4 (75%)^ | 4/7 (57%)^ | .55 | 1 (25%) | 3 (100%) | .11 |
| Muscle Relaxant* | 1/4 (25%)^ | 2/7 (29%)^ | .90 | 2 (50%) | 0 (0%) | .29 |
| Anticonvulsant* | 2/4 (50%)^ | 2/7 (29%)^ | .48 | 2 (50%) | 0 (0%) | .29 |
| Antidepressant/SSRI* | 2/4 (50%)^ | 2/7 (29%)^ | .48 | 0 (0%) | 2 (67%) | .14 |
| Narcotic Analgesic* | 0/4 (0%)^ | 1/7 (14%)^ | .43 | 0 (0%) | 1 (33%) | .43 |
| Anticoagulant* | 2/4 (50%)^ | 6/7 (86%)^ | .20 | 3 (75%) | 3 (100%) | .57 |
| Gastrointestinal* | 2/4 (50%)^ | 6/7 (86%)^ | .20 | 3 (75%) | 3 (100%) | .57 |
| Antihypertensive* | 1/4 (25%)^ | 5/7 (71%)^ | .14 | 4 (100%) | 1 (67%) | .14 |
| Antibiotic* | 1/4 (25%)^ | 3/7 (43%)^ | .55 | 2 (50%) | 1 (33%) | .63 |
| Nutritional Supplement* | 0/4 (0%)^ | 3/7 (43%)^ | .13 | 2 (50%) | 1(33%) | .63 |
| Alpha Blocker for Continence | 0/4 (0%)^ | 4/7 (57%)^ | .06 | 2 (50%) | 2(67%) | .63 |
| Hours Rehabilitation Services | 94.1; SD: 66.0^ ^n=3^ | 28.8; SD: 31.6^ ^n=6^ | .22 | 42.3; SD: 43.9 | 15.3; SD: 4.8 | .40 |
| # Informal Stimulation Interactions | 280.5; SD: 118.7 | 244.3; SD: 217.2 | .70 | 288.0; SD: 306.8 | 200.5 ± 104.2 | .61 |
| # Informal Talking Interactions | 78.8; SD: 33.7 | 69.9; SD: 58.8 | .73 | 84.0; SD: 81.8 | 55.8 ± 28.9 | .54 |
| # Informal Music Interactions | 37.3; SD: 23.4 | 29.0; SD: 23.5 | .53 | 24.5; SD: 26.2 | 33.5 ± 23.5 | .63 |
| # Other Informal Auditory Interactions | 20.0; SD: 13.0 | 18.6; SD: 25.9 | .90 | 27.5; SD: 35.1 | 9.8 ± 11.1 | .37 |
| # Informal Touching Interactions | 70.0; SD: 37.1 | 60.5; SD: 49.2 | .69 | 72.0; SD: 68.2 | 49.0 ± 25.3 | .55 |
| # Informal Visual Interactions | 20.8; SD: 15.1 | 24.3; SD: 27.3 | .77 | 26.0; SD: 38.0 | 22.5 ± 16.8 | .87 |
| # Informal Olfactory Interactions | 41.7; SD: 34.8 | 14.8; SD 10.7 | .12 | 11.3; SD: 10.8 | 18.3 ± 10.8 | .39 |
| # Other Stimuli Informal Interactions | 11.8; SD: 6.4 | 27.3; SD: 38.4 | .30 | 42.8; SD: 51.2 | 11.8 ± 13.6 | .29 |
| **AIS** = Abbreviated Injury Severity; **SAH** = Subarachnoid Hemorrhages; **SD** = Std Deviation; **SSRI =** Serotonin Reuptake Inhibitors; ^ missing data changes denominator; * White *vs* All Other; ** ≤ HS *vs* All Other; ^+^ moving vehicles *vs* All Other’; ^++^ MCS vs All Other | | | | | | |
