# Supplementary material for: Neural Connectivity Changes Facilitated by Familiar Auditory Sensory Training in Disordered Consciousness: A TBI Pilot Study
Source: Front Neurol. 2020 Oct 8;11:1027. doi: 10.3389/fneur.2020.01027 (PMC7578344; doi:10.3389/fneur.2020.01027)
Supplement: Supplementary file 6 [file Data_Sheet_6.docx]

| **Table SF1. Mean FA and MD Change using estimates with Standard Deviations (SD) (n = 4 per group)** | | | | | | | | | | | | | | |
| --- | --- | --- | --- | --- | --- | --- | --- | --- | --- | --- | --- | --- | --- | --- |
|  | **FAST FA** | | | **PLACEBO FA** | | | **FAST**  **vs Placebo**  **End – Base p** | **FAST MD (n = 4)** | | | **PLACEBO MD** | | | **FAST**  **vs PLACEBO**  **End – Base p** |
|  | **Mean FA (SD)** | | **End – Base p** | **Mean FA (SD)** | | **End –**  **Base p** |  | **Mean MD (SD)** | | **End – Base p** | **Mean MD (SD)** | | **End – Base p** |  |
|  | **Base** | **End** |  | **Base** | **End** |  |  | **Base** | **End** |  | **Base** | **End** |  |  |
| **Body of CC** | 0.36  (0.06) | 0.35  (0.05) | **0..07** | 0.39  (0.06) | 0.38  (0.05) | **0.0** | 0.23 | 0.00120  (0.00009) | 0.00124  (0.00016) | 0.13 | 0.00116  (0.00008) | 0.00122  (0.00005) | **0.00** | 0.60 |
| **Genu of CC** | 0.43  (0.10) | 0.42  (0.12) | 0.33 | 0.37  (0.02) | 0.36  (0.03) | .13 | 0.24 | 0.00105  (0.00005) | 0.00113  (0.00013) | 0.06 | 0.00111  (0.00009) | 0.00118  (0.00012) | **0.00** | 0.34 |
| **Splenium of CC** | 0.43  (0.06) | 0.43  (0.04) | 0.40 | 0.43  (0.12) | 0.39  (0.10) | **0.0** | 0.13 | 0.00101  (0.00005) | 0.00110  (0.00004) | **0.0** | 0.00112  (0.00019) | 0.00122  (0.00016) | **0.00** | 0.28 |
| **Left Ant IC Limb** | 0.41  (0.04) | 0.42  (0.05) | 0.40 | 0.35  (0.06) | 0.35  (0.03) | .40 | 0.34 | 0.00083  (0.00008) | 0.00084  (0.00010) | **0.33** | 0.00101  (0.00036) | 0.00099  (0.00028) | 0.40 | 0.43 |
| **Right Ant IC Limb** | 0.38  (0.04) | 0.39  (0.05) | 0.13 | 0.39  (0.04) | 0.37  (0.02) | **0.07** | **0.08** | 0.00085  (0.00009) | 0.00087  (0.00010) | **0.0** | 0.00091  (0.00015) | 0.00097  (0.00016) | **0.00** | **0.00** |
| **Left**  **IFOF** | 0.37  (0.01) | 0.34  (0.04) | **0.07** | 0.35  (0.02) | 0.31  (0.01) | **0.0** | 0.44 | 0.00088  (0.00003) | 0.00093  (0.00005) | **0.0** | 0.00094  (0.00007) | 0.00093  (0.00004) | 0.27 | **0.04** |
| **Right**  **IFOF** | 0.34  (0.08) | 0.35  (0.09) | 0.4 | 0.35  (0.03) | 0.33  (0.01) | **0.07** | 0.38 | 0.00101  (0.00017) | 0.00104  (0.00014) | 0.20 | 0.00090  (0.00003) | 0.00092  (0.00002) | **0.00** | 0.28 |
| **Left**  **ILF** | 0.38  (0.01) | 0.41  (0.01) | **0.0** | 0.38  (0.08) | 0.37  (0.08) | **0.0** | **0.00** | 0.00093  (0.00001) | 0.00091  (0.00005) | **0.06** | 0.00100  (0.00019) | 0.00095  (0.00027) | 0.27 | 0.37 |
| **Right**  **ILF** | 0.38  (0.05) | 0.41  (0.05) | **0.00** | 0.41  (0.03) | 0.36  (0.07) | **.20** | **0.01** | 0.00098  (0.00010) | 0.00094  (0.00010) | **0.0** | 0.00092  (0.00005) | 0.00107  (0.00033) | 0.20 | **0.06** |
| **Left**  **PTR** | 0.41  (0.05) | 0.39  (0.07) | 0.40 | 0.43  (0.02) | 0.40  (0.08) | .47 | 0.48 | 0.00100  (0.00040) | 0.00109  (0.00024) | 0.40 | 0.00103  (0.00021) | 0.00110  (0.00024) | 0.40 | 0.47 |
| **Right**  **PTR** | 0.40  (0.03) | 0.40  (0.02) | 0.40 | 0.36  (0.07) | 0.35  (0.08) | .20 | 0.24 | 0.00101  (0.00003) | 0.00106  (0.00005) | **0.0** | 0.00112  (0.00017) | 0.00122  (0.00022) | **0.00** | **0.04** |
| **Left**  **SFOF** | 0.35  (0.01) | 0.36  (0.02) | 0.20 | 0.35  (0.02) | 0.32  (0.01) | .20 | **0.00** | 0.00096  (0.00012) | 0.00095  (0.00014) | 0.27 | 0.00112  (0.00017) | 0.00122  (0.00022) | **0.00** | **0.00** |
| **Right SFOF** | 0.30  (0.03) | 0.37  (0.03) | **0.00** | 0.27  (0.09) | 0.33  (0.10) | **0.0** | **0.01** | 0.00096  (0.00012) | 0.00095  (0.00014) | 0.27 | 0.00112  (0.00017) | 0.00122  (0.00022) | **0.00** | **0.00** |
| **Left**  **SLF** | 0.41  (0.00) | 0.40  (0.02) | 0.40 | 0.39  (0.04) | 0.38  (0.03) | **0.0** | 0.18 | 0.00079  (0.00005) | 0.00079  (0.00005) | **.06** | 0.00079  (0.00003) | 0.00080  (0.00004) | 0.27 | 0.27 |
| **Right**  **SLF** | 0.41  (0.01) | 0.42  (0.01) | **0.00** | 0.35  (0.05) | 0.35  (0.05) | .33 | **0.01** | 0.00079  (0.00025) | 0.00077  (0.00069) | 0.40 | 0.00082  (0.00005) | 0.00083  (0.00003) | 0.20 | 0.43 |
| **Left**  **UF** | 0.33  (0.03) | 0.32  (0.04) | 0.47 | 0.31  (0.05) | 0.31  (0.06) | **.07** | 0.28 | 0.00093  (0.00015) | 0.00097  (0.00020) | 0.13 | 0.00091  (0.00006) | 0.00090  (0.00006) | 0.27 | 0.20 |
| **Right**  **UF** | 0.30  (0.04) | 0.32  (0.04) | **0.00** | 0.31  (0.05) | 0.30  (0.02) | 0.27 | 0.24 | 0.00093  (0.00015) | 0.00097  (0.00020) | 0.13 | 0.00094  (0.00006) | 0.00099  (0.00007) | **0.00** | 0.43 |
| **Left**  **AF** | 0.44  (0.04) | 0.44  (0.02) | 0.27 | 0.46  (0.05) | 0.44  (0.06) | **0.00** | **.06** | 0.00084  (0.00004) | 0.00072  (0.00017) | 0.33 | 0.00081  (0.00006) | 0.00084  (0.00006) | **0.00** | 0.18 |
| **Right**  **AF** | 0.39  (0.03) | 0.40  (0.03) | **0.0** | 0.39  (0.04) | 0.43  (0.07) | .40 | 0.48 | 0.00084  (0.00004) | 0.00085  (0.00006) | 0.27 | 0.00081  (0.00005) | 0.00082  (0.00006) | **0.04** | 0.45 |
| P = ordered p values; **Black** = significant p ≤ 0.05; **Gray shading =** p >0.05 & < 0.10; **Ant** = Anterior; **CC** = corpus callosum; **IC** = internal capsule; **IFOF & SFOF** = Inferior & Superior Fronto-Occipital Fasciculus; **ILF** = Inferior Longitudinal Fasciculus; **NA =** Not computed as n =1;  **PTR** = Posterior Thalamic Radiation; **SLF** = Superior Longitudinal Fasciculus; **UF** = Uncinated Fasciculus; **AF** = Arcuate Fasciculus | | | | | | | | | | | | | | |

| **Table SF2. FAST GROUP: Correlations Between Change In Estimated Z Values and Neurobehavioral Change** | | | | | | | | | | | |
| --- | --- | --- | --- | --- | --- | --- | --- | --- | --- | --- | --- |
|  | **FAST**  **Group** | **AN** | **DMN** | **LN** | **SN** | **AN-DMN** | **AN-LN** | **AN-SN** | **DMN-LN** | **DMN-SN** | **LN - SN** |
| **DOCS_TOTAL** | **PCC** | 0.52714 | -0.52794 | -0.617 | 0.6424 | 0.49887 | -0.59886 | 0.15744 | 0.7165 | -0.68707 | -0.26703 |
|  | **pvalues** | 0.4729 | 0.4721 | 0.383 | 0.3576 | 0.5011 | 0.4011 | 0.8426 | 0.2835 | 0.3129 | 0.733 |
|  | **n** | 4 | 4 | 4 | 4 | 4 | 4 | 4 | 4 | 4 | 4 |
| **DOCS Auditory-Language** | **PCC** | -0.29897 | 0.50557 | 0.36693 | -0.35669 | -0.16931 | 0.37018 | 0.13931 | -0.90727 | 0.91571 | 0.61959 |
|  | **pvalues** | 0.701 | 0.4944 | 0.6331 | 0.6433 | 0.8307 | 0.6298 | 0.8607 | 0.0927 | 0.0843 | 0.3804 |
|  | **n** | 4 | 4 | 4 | 4 | 4 | 4 | 4 | 4 | 4 | 4 |
| **CNC** | **PCC** | -0.50195 | 0.63286 | 0.81583 | -0.67982 | -0.58217 | 0.79539 | -0.44273 | -0.57581 | 0.46216 | -0.00804 |
|  | **pvalues** | 0.498 | 0.3671 | 0.1842 | 0.3202 | 0.4178 | 0.2046 | 0.5573 | 0.4242 | 0.5378 | 0.992 |
|  | **n** | 4 | 4 | 4 | 4 | 4 | 4 | 4 | 4 | 4 | 4 |
| **p =** ordered p values; **CNC** = Coma Near Coma Scale; **DOCS** = Disorders of Consciousness Scale-25; **DOCS_AUD** = DOCS-25 Auditory-Language Sub-scale Measures; **PCC =** Pearson Correlation Coefficient | | | | | | | | | | | |

| **Table SF3. PLACEBO GROUP: Correlations Between Change In Estimated Z Values And Neurobehavioral Change** | | | | | | | | | | | |
| --- | --- | --- | --- | --- | --- | --- | --- | --- | --- | --- | --- |
|  | **Placebo Group** | **AN** | **DMN** | **LN** | **SN** | **AN-DMN** | **AN-LN** | **AN-SN** | **DMN-LN** | **DMN-SN** | **LN - SN** |
| **DOCS_TOTAL** | **PCC** | 0.1752 | -0.48083 | 0.36427 | 0.04838 | -0.89062 | 0.07204 | 0.73231 | -0.51064 | 0.07677 | 0.64832 |
|  | **pvalues** | 0.8248 | 0.5192 | 0.6357 | 0.9516 | 0.1094 | 0.928 | 0.2677 | 0.4894 | 0.9232 | 0.3517 |
|  | **n** | 4 | 4 | 4 | 4 | 4 | 4 | 4 | 4 | 4 | 4 |
| **DOCS Auditory-Language** | **PCC** | 0.33502 | -0.54779 | 0.58118 | -0.31483 | -0.94722 | 0.22888 | 0.86454 | -0.78771 | 0.17789 | 0.84868 |
|  | **pvalues** | 0.665 | 0.4522 | 0.4188 | 0.6852 | 0.0528 | 0.7711 | 0.1355 | 0.2123 | 0.8221 | 0.1513 |
|  | **n** | 4 | 4 | 4 | 4 | 4 | 4 | 4 | 4 | 4 | 4 |
| **CNC** | **PCC** | -0.19754 | -0.00083 | -0.19461 | -0.45659 | 0.47663 | -0.1351 | -0.51239 | -0.01011 | -0.22995 | -0.32968 |
|  | **pvalues** | 0.8025 | 0.9992 | 0.8054 | 0.5434 | 0.5234 | 0.8649 | 0.4876 | 0.9899 | 0.77 | 0.6703 |
|  | **n** | 4 | 4 | 4 | 4 | 4 | 4 | 4 | 4 | 4 | 4 |
| **p =** ordered p values; **CNC** = Coma Near Coma Scale; **DOCS** = Disorders of Consciousness Scale-25; **DOCS_AUD** = DOCS-25 Auditory-Language Sub-scale Measures; **PCC =** Pearson Correlation Coefficient | | | | | | | | | | | |

**Table SF4. Baseline Mean FA Values by Intervention Groups**

|  | Group Assignment | N | Mean | Std. Deviation | Std. Error Mean |
| --- | --- | --- | --- | --- | --- |
| Baseline FA Body of Corpus Callosum | Placebo | 4 | .390000000 | .0547722558 | .0273861279 |
|  | FAST | 4 | .357497500 | .0580645161 | .0290322580 |
| Baseline FA Genu of Corpus Callosum | Placebo | 4 | .373635000 | .0133851024 | .0066925512 |
|  | FAST | 4 | .425845000 | .0992419658 | .0496209829 |
| Baseline FA Splenium of Corpus Callosum | Placebo | 4 | .432357500 | .1143451912 | .0571725956 |
|  | FAST | 4 | .432407500 | .0619432048 | .0309716024 |
| Baseline FA of Left Anterior Internal Capsule | Placebo | 4 | .351840000 | .0510996243 | .0255498121 |
|  | FAST | 4 | .409952500 | .0399359899 | .0199679950 |
| Baseline FA of Right Anterior Internal Cap. | Placebo | 4 | .389200000 | .0307163919 | .0153581960 |
|  | FAST | 4 | .378900000 | .0356517667 | .0178258833 |
| Baseline FA of Left Inferior Occipital Fasciculus | Placebo | 4 | .346410000 | .0129756002 | .0064878001 |
|  | FAST | 4 | .370470000 | .0073845514 | .0036922757 |
| Baseline FA of Right Inferior Occipital Fasciculus | Placebo | 4 | .350057500 | .0268433398 | .0134216699 |
|  | FAST | 4 | .343542500 | .0839282171 | .0419641085 |
| Baseline FA of Left Inferior Longitudinal Fasciculus | Placebo | 4 | .381745000 | .0687646443 | .0343823221 |
|  | FAST | 4 | .381662500 | .0144486548 | .0072243274 |
| Baseline FA of Right Inferior Longitudinal Fasciculus | Placebo | 4 | .409482500 | .0453219508 | .0226609754 |
|  | FAST | 4 | .383172500 | .0494198397 | .0247099199 |
| Baseline FA of Left Post Thalamic Radiation | Placebo | 4 | .425447500 | .2725726393 | .1362863196 |
|  | FAST | 4 | .405270000 | .0511202380 | .0255601190 |
| Baseline FA of Right Post Thalamic Radiation | Placebo | 4 | .357500000 | .0710992250 | .0355496125 |
|  | FAST | 4 | .398507500 | .0258324400 | .0129162200 |
| Baseline FA of Left Sup Fronto-Occipital Fasciculus | Placebo | 4 | .352560000 | .0000000000 | .0000000000 |
|  | FAST | 4 | .350537500 | .0061841808 | .0030920904 |
| Baseline FA of Right Superior Fronto-Occipital Fasciculus | Placebo | 4 | .274487500 | .0560810677 | .0280405339 |
|  | FAST | 4 | .298747500 | .0262302654 | .0131151327 |
| Baseline FA of Left Superior Longitudinal Fasciculus | Placebo | 4 | .392747500 | .0353913976 | .0176956988 |
|  | FAST | 4 | .405807500 | .0023544904 | .0011772452 |
| Baseline FA of Right Superior Longitudinal Fasciculus | Placebo | 4 | .353362500 | .0470583052 | .0235291526 |
|  | FAST | 4 | .405895000 | .0087467232 | .0043733616 |
| Baseline FA of Left Uncinate Fasciculus | Placebo | 4 | .313627500 | .0420530573 | .0210265286 |
|  | FAST | 4 | .325117500 | .0334699132 | .0167349566 |
| Baseline FA of Right Uncinate Fasciculus | Placebo | 4 | .309987500 | .0190782832 | .0095391416 |
|  | FAST | 4 | .298207500 | .0429647098 | .0214823549 |
| Baseline FA of Left Arcuate Fasciculus | Placebo | 4 | .457987500 | .0437541163 | .0218770581 |
|  | FAST | 4 | .436277500 | .0353281629 | .0176640814 |
| Baseline FA of Right Arcuate Fasciculus | Placebo | 4 | .431507500 | .0477563039 | .0238781519 |
|  | FAST | 4 | .389762500 | .0292457659 | .0146228830 |

**Table SF5. Comparability of Baseline Mean FA Values: FAST *versus* Placebo Groups**

| **Independent Samples Test** | | | | | | | | | | | |
| --- | --- | --- | --- | --- | --- | --- | --- | --- | --- | --- | --- |
|  | | Levene's Test for  Equality of Variances | | t-test for Equality of Means | | | | | | |  |
|  |  | F | Sig. | t | df | Sig. (2-tailed) | Mean Difference | Std. Error Difference | 95% Confidence Interval of the Difference | |  |
|  |  |  |  |  |  |  |  |  | Lower | Upper |  |
| Baseline FA Body of Corpus Callosum | = variances assumed | .004 | .953 | .814 | 6 | .447 | .0325025000 | .0399108006 | -.0651557110 | .1301607110 |  |
|  | = variances not assumed |  |  | .814 | 5.980 | .447 | .0325025000 | .0399108006 | -.0652362412 | .1302412412 |  |
| Baseline FA Genu of Corpus Callosum | = variances assumed | 5.034 | .066 | -1.043 | 6 | .337 | -.0522100000 | .0500702725 | -.1747275431 | .0703075431 |  |
|  | = variances not assumed |  |  | -1.043 | 3.109 | .371 | -.0522100000 | .0500702725 | -.2084386941 | .1040186941 |  |
| Baseline FA Splenium of Corpus Callosum. | = variances assumed | 1.558 | .259 | -.001 | 6 | .999 | -.0000500000 | .0650226564 | -.1591547085 | .1590547085 |  |
|  | = variances not assumed |  |  | -.001 | 4.621 | .999 | -.0000500000 | .0650226564 | -.1714028150 | .1713028150 |  |
| Baseline FA of Left Anterior Internal Capsule | = variances assumed | .104 | .758 | -1.792 | 6 | .123 | -.0581125000 | .0324270523 | -.1374586386 | .0212336386 |  |
|  | = variances not assumed |  |  | -1.792 | 5.669 | .126 | -.0581125000 | .0324270523 | -.1385951989 | .0223701989 |  |
| Baseline FA of Right Anterior Internal Cap. | = variances assumed | .006 | .939 | .438 | 6 | .677 | .0103000000 | .0235294773 | -.0472745568 | .0678745568 |  |
|  | = variances not assumed |  |  | .438 | 5.872 | .677 | .0103000000 | .0235294773 | -.0475812619 | .0681812619 |  |
| Baseline FA of Left Inferior Occipital Fasciculus | = variances assumed | .563 | .481 | -3.223 | 6 | .018 | -.0240600000 | .0074648811 | -.0423259061 | -.0057940939 |  |
|  | = variances not assumed |  |  | -3.223 | 4.759 | .025 | -.0240600000 | .0074648811 | -.0435453251 | -.0045746749 |  |
| Baseline FA of Right Inferior Occipital Fasciculus | = variances assumed | 2.183 | .190 | .148 | 6 | .887 | .0065150000 | .0440582300 | -.1012916051 | .1143216051 |  |
|  | = variances not assumed |  |  | .148 | 3.607 | .890 | .0065150000 | .0440582300 | -.1212435134 | .1342735134 |  |
| Baseline FA of Left Inferior Longitudinal Fasciculus | = variances assumed | 2.155 | .192 | .002 | 6 | .998 | .0000825000 | .0351331038 | -.0858851080 | .0860501080 |  |
|  | = variances not assumed |  |  | .002 | 3.264 | .998 | .0000825000 | .0351331038 | -.1067744398 | .1069394398 |  |
| Baseline FA of Right Inferior Longitudinal Fasciculus | = variances assumed | .004 | .952 | .785 | 6 | .462 | .0263100000 | .0335275998 | -.0557290812 | .1083490812 |  |
|  | = variances not assumed |  |  | .785 | 5.956 | .463 | .0263100000 | .0335275998 | -.0558775722 | .1084975722 |  |
| Baseline FA of Left Post Thalamic Radiation | = variances assumed | 6.334 | .045 | .146 | 6 | .889 | .0201775000 | .1386624701 | -.3191173413 | .3594723413 |  |
|  | = variances not assumed |  |  | .146 | 3.211 | .893 | .0201775000 | .1386624701 | -.4051637749 | .4455187749 |  |
| Baseline FA of Right Post Thalamic Radiation | = variances assumed | 1.556 | .259 | -1.084 | 6 | .320 | -.0410075000 | .0378233220 | -.1335578349 | .0515428349 |  |
|  | = variances not assumed |  |  | -1.084 | 3.778 | .343 | -.0410075000 | .0378233220 | -.1484950325 | .0664800325 |  |
| Baseline FA of Left Sup Fronto-Occipital Fasciculus | = variances assumed | 7.499 | .034 | .654 | 6 | .537 | .0020225000 | .0030920904 | -.0055435726 | .0095885726 |  |
|  | = variances not assumed |  |  | .654 | 3.000 | .560 | .0020225000 | .0030920904 | -.0078179116 | .0118629116 |  |
| Baseline FA of Right Superior Fronto-Occipital Fasciculus | = variances assumed | .625 | .459 | -.784 | 6 | .463 | -.0242600000 | .0309560696 | -.1000067736 | .0514867736 |  |
|  | = variances not assumed |  |  | -.784 | 4.253 | .475 | -.0242600000 | .0309560696 | -.1082311916 | .0597111916 |  |
| Baseline FA of Left Superior Longitudinal Fasciculus. | = variances assumed | 3.421 | .114 | -.736 | 6 | .489 | -.0130600000 | .0177348150 | -.0564555290 | .0303355290 |  |
|  | = variances not assumed |  |  | -.736 | 3.027 | .514 | -.0130600000 | .0177348150 | -.0692209831 | .0431009831 |  |
| Baseline FA of Right Superior Longitudinal Fasciculus | = variances assumed | 4.099 | .089 | -2.195 | 6 | .071 | -.0525325000 | .0239321398 | -.1110923365 | .0060273365 |  |
|  | = variances not assumed |  |  | -2.195 | 3.207 | .110 | -.0525325000 | .0239321398 | -.1259878279 | .0209228279 |  |
| Baseline FA of Left Uncinate Fasciculus | = variances assumed | .027 | .875 | -.428 | 6 | .684 | -.0114900000 | .0268732893 | -.0772465701 | .0542665701 |  |
|  | = variances not assumed |  |  | -.428 | 5.712 | .685 | -.0114900000 | .0268732893 | -.0780576184 | .0550776184 |  |
| Baseline FA of Right Uncinate Fasciculus | = variances assumed | 1.191 | .317 | .501 | 6 | .634 | .0117800000 | .0235050377 | -.0457347552 | .0692947552 |  |
|  | = variances not assumed |  |  | .501 | 4.139 | .642 | .0117800000 | .0235050377 | -.0526263998 | .0761863998 |  |
| Baseline FA of Left Arcuate Fasciculus | = variances assumed | .000 | .990 | .772 | 6 | .469 | .0217100000 | .0281180626 | -.0470924207 | .0905124207 |  |
|  | = variances not assumed |  |  | .772 | 5.745 | .471 | .0217100000 | .0281180626 | -.0478396642 | .0912596642 |  |
| Baseline FA of Right Arcuate Fasciculus | = variances assumed | .477 | .515 | 1.491 | 6 | .187 | .0417450000 | .0279999080 | -.0267683066 | .1102583066 |  |
|  | = variances not assumed |  |  | 1.491 | 4.973 | .196 | .0417450000 | .0279999080 | -.0303499949 | .1138399949 |  |

**Table SF6. BASELINE MEAN MD VALUES by Intervention Groups**

|  | Groups | N | Mean | Std. Deviation | Std. Error Mean |
| --- | --- | --- | --- | --- | --- |
| Baseline MD of Body of Corpus Callosum | Placebo | 4 | .001158750 | .0000796668 | .0000398334 |
|  | FAST | 4 | .001200000 | .0000816497 | .0000408248 |
| Baseline MD of Genu of Corpus Callosum | Placebo | 4 | .001114000 | .0000853190 | .0000426595 |
|  | FAST | 4 | .001045000 | .0000481110 | .0000240555 |
| Baseline MD of Splenium Corpus Callosum | Placebo | 4 | .001123500 | .0001919818 | .0000959909 |
|  | FAST | 4 | .001012750 | .0000514482 | .0000257241 |
| Baseline MD of Left Anterior Internal Capsule | Placebo | 4 | .001008000 | .0003624353 | .0001812176 |
|  | FAST | 4 | .000826750 | .0000831319 | .0000415660 |
| Baseline MD of Right Anterior Internal Capsule | Placebo | 4 | .002845000 | .0038393446 | .0019196723 |
|  | FAST | 4 | .000847250 | .0000886430 | .0000443215 |
| Baseline MD of Left Inferior Occipital Fasciculus | Placebo | 4 | .000925000 | .0000500000 | .0000250000 |
|  | FAST | 4 | .000882000 | .0000320416 | .0000160208 |
| Baseline MD of Right Inferior Occipital Fasciculus | Placebo | 4 | .000892500 | .0000330404 | .0000165202 |
|  | FAST | 4 | .001014000 | .0001659900 | .0000829950 |
| Baseline MD of Left Inferior Longitudinal Fasciculus | Placebo | 4 | .000917250 | .0001807436 | .0000903718 |
|  | FAST | 4 | .000930000 | .0000069761 | .0000034881 |
| Baseline MD of Right Inferior Longitudinal Fasciculus | Placebo | 4 | .000916250 | .0000502950 | .0000251475 |
|  | FAST | 4 | .000980750 | .0000963756 | .0000481878 |
| Baseline MD of Left Post Thalamic Radiation | Placebo | 4 | .001032750 | .0002085735 | .0001042868 |
|  | FAST | 4 | .000996500 | .0004043278 | .0002021639 |
| Baseline MD of Right Post Thalamic Radiation | Placebo | 4 | .001119000 | .0001732763 | .0000866381 |
|  | FAST | 4 | .001008000 | .0000269815 | .0000134907 |
| Baseline MD of Left Superior Fronto-Occipital Fasciculus | Placebo | 4 | .001119000 | .0001732763 | .0000866381 |
|  | FAST | 4 | .000958000 | .0001154903 | .0000577451 |
| Baseline MD of Right Superior Front-Occipital Fasciculus | Placebo | 4 | .001119000 | .0001732763 | .0000866381 |
|  | FAST | 4 | .000958000 | .0001154903 | .0000577451 |
| Baseline MD of Left Superior Longitudinal Fasciculus | Placebo | 4 | .000785500 | .0000324808 | .0000162404 |
|  | FAST | 4 | .000791250 | .0000487810 | .0000243905 |
| Baseline MD of Right Superior Longitudinal Fasciculus | Placebo | 4 | .000818000 | .0000511924 | .0000255962 |
|  | FAST | 4 | .000786250 | .0002521380 | .0001260690 |
| Baseline MD of Left Uncinate Fasciculus | Placebo | 4 | .000913750 | .0000555780 | .0000277890 |
|  | FAST | 4 | .000933000 | .0001540411 | .0000770206 |
| Baseline MD of Right Uncinate Fasciculus | Placebo | 4 | .000938500 | .0000613433 | .0000306716 |
|  | FAST | 4 | .000933000 | .0001540411 | .0000770206 |
| Baseline MD of Left Arcuate Fasciculus | Placebo | 4 | .000814250 | .0000584601 | .0000292301 |
|  | FAST | 4 | .000789750 | .0000122848 | .0000061424 |
| Baseline MD of Right Arcuate Fasciculus | Placebo | 4 | .000814000 | .0000453725 | .0000226863 |
|  | FAST | 3 | .000843667 | .0000381750 | .0000220404 |

**Table SF7. Comparability of Baseline Mean FA Values: FAST *versus* Placebo**

|  | | | | | | | | | | | |
| --- | --- | --- | --- | --- | --- | --- | --- | --- | --- | --- | --- |
|  | | Levene's Test for  Equality of Variances | | t-test for Equality of Means | | | | | | |  |
|  |  | F | Sig. | t | df | Sig.  (2-tailed) | Mean  Difference | Std. Error  Difference | 95% Confidence Interval of the Difference | |  |
|  |  |  |  |  |  |  |  |  | Lower | Upper |  |
| Baseline MD of Body of Corpus Callos. | = variances assumed | .075 | .794 | -.723 | 6 | .497 | -.0000412500 | .0000570383 | -.0001808176 | .0000983176 |  |
|  | = variances not assumed |  |  | -.723 | 5.996 | .497 | -.0000412500 | .0000570383 | -.0001808381 | .0000983381 |  |
| Baseline MD of Genu of Corpus Callos. | = variances assumed | .861 | .389 | 1.409 | 6 | .209 | .0000690000 | .0000489745 | -.0000508362 | .0001888362 |  |
|  | = variances not assumed |  |  | 1.409 | 4.733 | .221 | .0000690000 | .0000489745 | -.0000590621 | .0001970621 |  |
| Baseline MD of Splenium Corpus Callos. | = variances assumed | 8.695 | .026 | 1.114 | 6 | .308 | .0001107500 | .0000993780 | -.0001324191 | .0003539191 |  |
|  | = variances not assumed |  |  | 1.114 | 3.429 | .337 | .0001107500 | .0000993780 | -.0001842805 | .0004057805 |  |
| Baseline MD of Left Anterior Internal Cap. | = variances assumed | 4.066 | .090 | .975 | 6 | .367 | .0001812500 | .0001859235 | -.0002736885 | .0006361885 |  |
|  | = variances not assumed |  |  | .975 | 3.315 | .395 | .0001812500 | .0001859235 | -.0003798894 | .0007423894 |  |
| Baseline MD of Right Anterior Internal Cap. | = variances assumed | 8.600 | .026 | 1.040 | 6 | .338 | .0019977500 | .0019201839 | -.0027007706 | .0066962706 |  |
|  | = variances not assumed |  |  | 1.040 | 3.003 | .375 | .0019977500 | .0019201839 | -.0041094520 | .0081049520 |  |
| Baseline MD of Left Inferior Occip Fascic. | = variances assumed | .889 | .382 | 1.448 | 6 | .198 | .0000430000 | .0000296929 | -.0000296558 | .0001156558 |  |
|  | = variances not assumed |  |  | 1.448 | 5.108 | .206 | .0000430000 | .0000296929 | -.0000328433 | .0001188433 |  |
| Baseline MD of Right Inferior Occip Fascic. | = variances assumed | 3.487 | .111 | -1.436 | 6 | .201 | -.0001215000 | .0000846232 | -.0003285655 | .0000855655 |  |
|  | = variances not assumed |  |  | -1.436 | 3.237 | .240 | -.0001215000 | .0000846232 | -.0003799760 | .0001369760 |  |
| Baseline MD of Left Inferior Longitud Fascic. | = variances assumed | 4.796 | .071 | -.141 | 6 | .893 | -.0000127500 | .0000904391 | -.0002340465 | .0002085465 |  |
|  | = variances not assumed |  |  | -.141 | 3.009 | .897 | -.0000127500 | .0000904391 | -.0003000845 | .0002745845 |  |
| Baseline MD of Right Inferior Longitud Fascic. | = variances assumed | 1.066 | .342 | -1.187 | 6 | .280 | -.0000645000 | .0000543549 | -.0001975017 | .0000685017 |  |
|  | = variances not assumed |  |  | -1.187 | 4.521 | .294 | -.0000645000 | .0000543549 | -.0002087936 | .0000797936 |  |
| Baseline MD of Left Post Thalamic Radiat. | = variances assumed | .853 | .391 | .159 | 6 | .879 | .0000362500 | .0002274774 | -.0005203672 | .0005928672 |  |
|  | = variances not assumed |  |  | .159 | 4.491 | .880 | .0000362500 | .0002274774 | -.0005690048 | .0006415048 |  |
| Baseline MD of Right Post Thalamic Radiat. | = variances assumed | 14.153 | .009 | 1.266 | 6 | .252 | .0001110000 | .0000876822 | -.0001035506 | .0003255506 |  |
|  | = variances not assumed |  |  | 1.266 | 3.145 | .291 | .0001110000 | .0000876822 | -.0001608868 | .0003828868 |  |
| Baseline MD of Left Sup Fronto-Occip Fascic. | = variances assumed | 1.553 | .259 | 1.546 | 6 | .173 | .0001610000 | .0001041185 | -.0000937688 | .0004157688 |  |
|  | = variances not assumed |  |  | 1.546 | 5.226 | .180 | .0001610000 | .0001041185 | -.0001031996 | .0004251996 |  |
| Baseline MD of Right Sup Front-Occip Fascic. | = variances assumed | 1.553 | .259 | 1.546 | 6 | .173 | .0001610000 | .0001041185 | -.0000937688 | .0004157688 |  |
|  | = variances not assumed |  |  | 1.546 | 5.226 | .180 | .0001610000 | .0001041185 | -.0001031996 | .0004251996 |  |
| Baseline MD of Left Sup Longitud Fascic. | = variances assumed | .255 | .632 | -.196 | 6 | .851 | -.0000057500 | .0000293027 | -.0000774510 | .0000659510 |  |
|  | = variances not assumed |  |  | -.196 | 5.223 | .852 | -.0000057500 | .0000293027 | -.0000801172 | .0000686172 |  |
| Baseline MD of Right Sup Longitud Fascic. | = variances assumed | 3.247 | .122 | .247 | 6 | .813 | .0000317500 | .0001286412 | -.0002830237 | .0003465237 |  |
|  | = variances not assumed |  |  | .247 | 3.247 | .820 | .0000317500 | .0001286412 | -.0003605826 | .0004240826 |  |
| Baseline MD of Left Uncinate Fascic. | = variances assumed | 2.085 | .199 | -.235 | 6 | .822 | -.0000192500 | .0000818804 | -.0002196041 | .0001811041 |  |
|  | = variances not assumed |  |  | -.235 | 3.768 | .826 | -.0000192500 | .0000818804 | -.0002522133 | .0002137133 |  |
| Baseline MD of Right Uncinate Fascic. | = variances assumed | 1.751 | .234 | .066 | 6 | .949 | .0000055000 | .0000829031 | -.0001973565 | .0002083565 |  |
|  | = variances not assumed |  |  | .066 | 3.928 | .950 | .0000055000 | .0000829031 | -.0002263454 | .0002373454 |  |
| Baseline MD of Left Acuate Fascic. | = variances assumed | 3.523 | .110 | .820 | 6 | .443 | .0000245000 | .0000298685 | -.0000485855 | .0000975855 |  |
|  | = variances not assumed |  |  | .820 | 3.264 | .468 | .0000245000 | .0000298685 | -.0000663438 | .0001153438 |  |
| Baseline MD of Right Acuate Fascic. | = variances assumed | .114 | .749 | -.911 | 5 | .404 | -.0000296667 | .0000325665 | -.0001133816 | .0000540482 |  |
|  | = variances not assumed |  |  | -.938 | 4.852 | .393 | -.0000296667 | .0000316298 | -.0001117253 | .0000523920 |  |


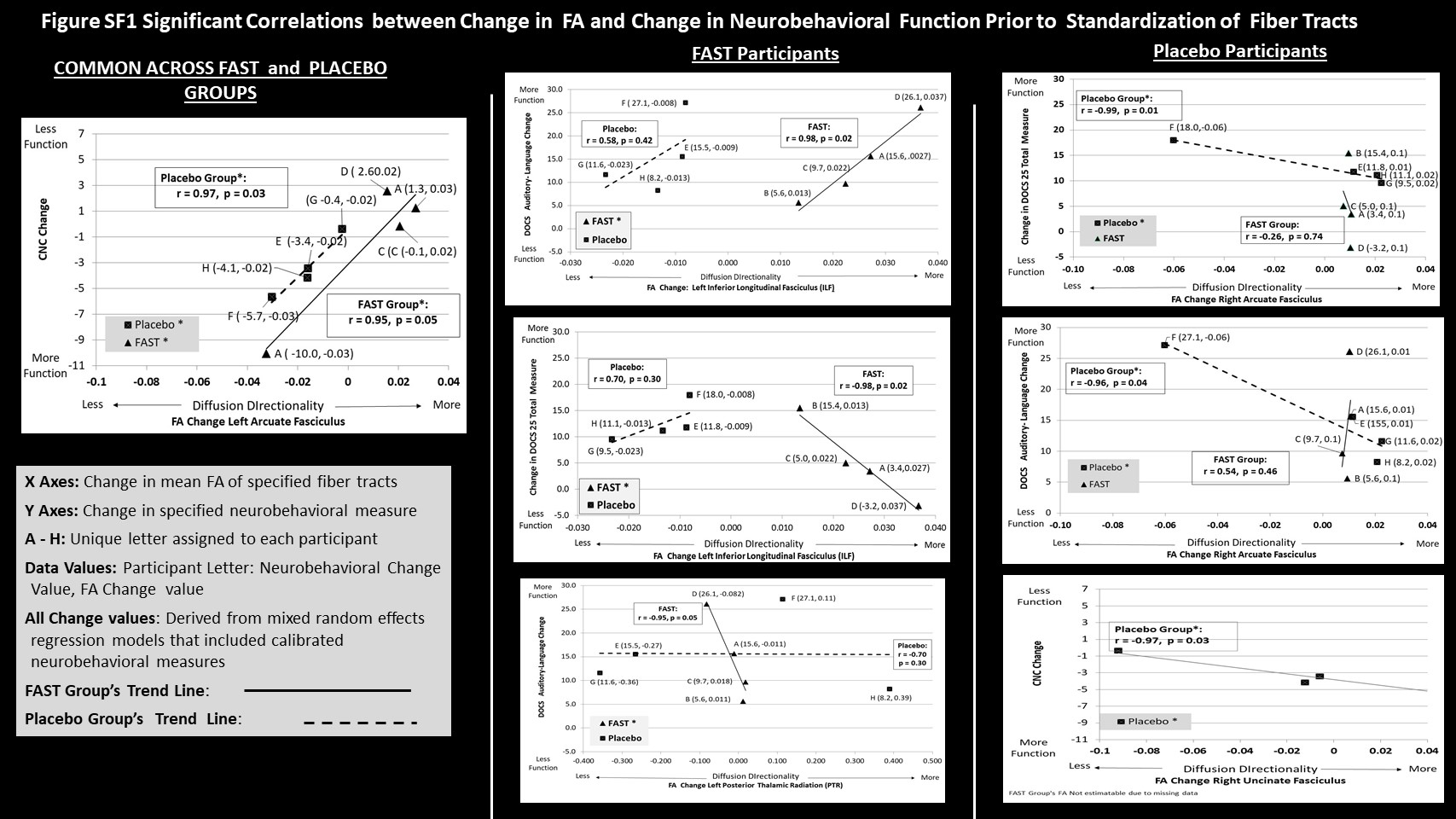


**DOCS 25**= Disorders of Consciousness Scale-2 **Auditory-Lang.** = DOCS Auditory-Language Sub-scale; **CNC** = Coma Near Coma
